# Exploratory Data Analysis of the In Vitro Effects of Novel Hydrazide-Hydrazone Antioxidants in the Context of In Silico Predictors

**DOI:** 10.3390/antiox14050566

**Published:** 2025-05-08

**Authors:** Yordan Yordanov, Virginia Tzankova, Denitsa Stefanova, Maya Georgieva, Diana Tzankova

**Affiliations:** 1 Department of Pharmacology, Pharmacotherapy and Toxicology, Faculty of Pharmacy, Medical University—Sofia, 2 Dunav Str., 1000 Sofia, Bulgariadenitsa.stefanova@pharmfac.mu-sofia.bg (D.S.); 2 Department of Pharmaceutical Chemistry, Faculty of Pharmacy, Medical University—Sofia, 2 Dunav Str., 1000 Sofia, Bulgaria

**Keywords:** N-pyrrolyl hydrazones, antioxidant, in vitro, in silico, multiple factor analysis, clustering, variable selection, structure–activity relationship, cheminformatics

## Abstract

Substantial in vitro experimental data have been produced about the safety, antioxidant, neuro- and hepatoprotective effects of a series of recently synthesized N-pyrrolyl hydrazide-hydrazones (compounds **5**, **5a**–**5g**). However, compound activity across multiple assays varies and it is challenging to elucidate the favorable physicochemical characteristics of the studied compounds and guide further lead optimization. The aim of the current study is to apply exploratory data analysis in order to profile the biological effects of the novel hydrazide-hydrazones, gain insights related to their mechanisms of action in the context of in silico predictions and identify key predictor–outcome relationships. We collected a dataset from available in vitro studies of compounds **5**, **5a**–**5g**. It included cytotoxicity values, protection against hydrogen peroxide-induced damage in HepG2 and SH-SY5Y cells, two radical scavenging assays and a hemolysis assay across a range of treatment concentrations. SwissADME-based predictions of chemometric and ADME parameters and pro-oxidant enzyme docking data were generated to provide context for the interpretation of in vitro outcome patterns and identify causal relationships. Multiple factor analysis (MFA), followed by hierarchical clustering on principal components (HCPC), was applied to profile compounds’ biological behavior. This revealed that differences in the number of H-bond donors, in the permeability coefficient and in the docking scores to two pro-oxidant enzymes could aid in explaining the effects of compounds with similar in vitro profiles. HCPC differentiated **5a** as mostly neuroprotective, **5** and **5d** as hepatoprotective radical scavengers, **5g** with higher docking affinity to 5-lipoxygenase (5-LOX) and myeloperoxidase (MPO) and **5b**, **5c** and **5f** as having less H-bond donors and variable in vitro activity. The consensus application of three variable selection approaches based on standard lasso regression, robust penalized regression and random forest confirmed the relationships between some in vitro outcomes and LogP, pan-assay interference (PAINS) alerts, 5-LOX allosteric site docking and H-bond donor numbers. The exploratory analysis of the combined in vitro and in silico dataset provides useful insights which could help explain the major drivers behind the experimental results. It can be informative in the design of new, improved members of the series of novel N-pyrrolyl hydrazide-hydrazones with better neuroprotective potential and less side effects.

## 1. Introduction

Oxidative stress, continuous low-grade inflammation and tissue damage are remarkable in neurodegenerative conditions [1,2,3]. Inflammation outside the central nervous system (CNS) also impacts neurodegenerative disorders due to the complex interplay between systemic inflammation and neuroinflammation [4,5,6]. Thus, the long-term treatment strategy could benefit from mitigating such conditions, characterized by oxidative stress and inflammation, which occur simultaneously and potentiate each other [7,8,9,10,11,12,13,14,15].

Tissue damage and degeneration in inflammatory conditions is predominantly caused by the production of pro-oxidant molecules by phagocytes, catalyzed by NADPH oxidase (NOX) [16] and neutrophil myeloperoxidase (MPO) [17,18] enzymes. Cyclooxygenase [19] and lipoxygenase [20,21,22] enzymes also modulate reactive oxygen species (ROS) generation indirectly or directly and further amplify chronic inflammation [23,24] and endothelial dysfunction [25,26,27]. Alcoholic and non-alcoholic steatohepatitis is also a major contributor to systemic oxidative stress and is significantly affected by Cyp2E1 decoupling [13].

To date, the established pharmacotherapeutic approaches are focused on suppressing inflammation and controlling concurrent symptoms [28]. However, these therapies are not devoid of toxic side effects due to the physiological relevance of their pharmacodynamic targets [28]. Biologics therapies are selective, but costly, and are not devoid of toxic side effects [29]. The most researched approach for reducing oxidative damage is the use of natural antioxidants which act predominantly by scavenging ROS and indirectly by stimulating the Keap1/Nrf2/ARE pathway [30,31,32,33,34]. However, clinical studies with natural antioxidants have so far failed to demonstrate efficacy [35].

An improved strategy for handling chronic oxidative damage could be the development of multitarget compounds, which are both radical scavengers and inhibitors of endogenous ROS production. However, up to this moment, no authorized drug decreases endogenous oxidative species production as a primary mechanism [36]. A clinical trial involving patients with mutations in leukotriene A4 hydrolase undergoing coronary angiography showed that the 5-LOX inhibitor zileuton improves endothelial function [37]. However, its application is limited by its hepatotoxicity [38]. Clomethiazole, a sedative hypnotic drug and Cyp2E1 inhibitor, is effective in patients with alcoholic liver disease [39,40,41] and may provide therapeutic benefit in the treatment of acute ischemic stroke [42], although its application is also limited by its unfavorable safety profile [43]. Allopurinol, a xanthine oxidase inhibitor, may reduce ROS-related cardiovascular disease complications at low doses [44]. Drugs for other therapeutic indications with NOX-inhibiting ‘side effects’ are calcium channel blockers [45] and dextromethorphan [46]. Their application as neuroprotectors is also limited by the risk of toxicity. The natural product apocynin exhibits NOX-inhibiting activity but has failed to show efficacy in clinical trials [47]. Setanaxib, a NOX inhibitor, has so far shown promising results in the treatment of pulmonary fibrosis, diabetes and kidney disease in clinical trials [48]. Melatonin has been known for its MPO-inhibiting activity and was recently proposed as a safe antioxidant [49].

**Figure 1:**
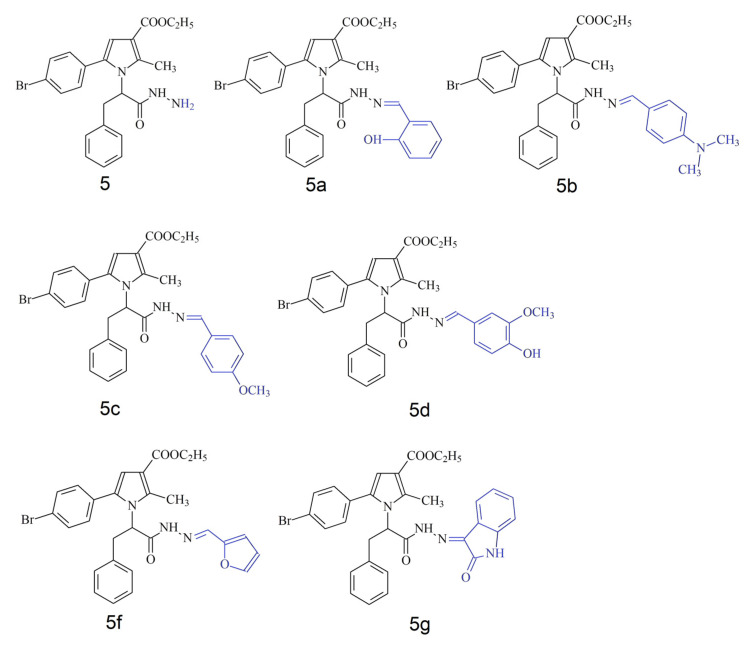
Chemical structures of **5**, **5a**–**5g** series of N-pyrrolyl hydrazide-hydrazones. Variable part of molecule presented in blue.

An underexplored class of novel N-pyrrolyl hydrazide-hydrazones (Figure 1) have shown promise as antioxidants in initial in vitro studies. Their synthesis has been described by Bijev and Georieva [50]. This small group of compounds is characterized by favorable bioavailability predictions [51], stability [52] and general enzyme-inhibiting propensities [51]. It has been experimentally shown that they are strong in vitro radical scavengers and cytoprotectors in models of hydrogen peroxide-damaged HepG2 cells [53] and SH-SY5Y cells [54]. In vivo studies with such compounds have shown acute median lethal doses in mice (LD50) in the typical range seen for most established therapeutic drugs on the market [55].

Previous studies of different hydrazone derivatives have progressed to ranking compounds by their diverse in vitro activities and interpreting those ranks in the context of SwissADME [56] predictions and docking scores, yet they fall short of integrating these datasets within a unified multivariate framework [57,58,59,60].

The current study aims to collect all relevant in vitro data about the series of novel N-pyrrolyl hydrazide-hydrazone compounds [53] in a single dataset, integrate it with in silico predictors, profile and group them based on similarity, identify the properties which are responsible for their diverse effects in the context of in silico predictions and use this to inform further lead optimization.

## 2. Materials and Methods

### 2.1. Dataset Collection

A detailed description of the in vitro and in silico data used, as well as the methods applied for generating the data, is available in Supplementary Materials S1–S3. All data analyses were performed on the statistical programming language R (version 4.4.3) [61] and the RStudio integrated development environment (version 2024.12.1.563) [62]. Complete R session information and all package version numbers can also be found in Supplementary Material S1.

A typical feature of the collected in vitro data is that they represent readings after treatment with more than one concentration and more than one replicate per concentration. The number of concentrations for each assay varies, and most concentrations are in the range between 1 and 100 µM. This part of the dataset consists of 7 unique model–endpoint combinations and 16 unique concentrations, some of which coincide across models and endpoints, while others differ. Unlike in vitro data, the in silico part of the dataset (except for docking scores) consists of single predicted output values, representing mostly predicted constants as mw, TPSA, LogP, bioavailability score, etc., which do not vary with concentration. They are generated based on the sole input being the chemical structure of compounds in an appropriate file format [56]. Many of the in silico parameters represent redundant information due to some of them being the output of models, predicting the same or a related physicochemical or medical chemistry-related parameter. All endpoints were included in order to not disregard the identification of weak associations due to the small observation size and the effects of outliers. Most of these parameters are represented as numeric values, but some are nominal (i.e., P-gp substrate) or ordinal (i.e., LogS water solubility class).

### 2.2. Combined In Vitro and In Silico Dataset Preprocessing

In order to perform exploratory analyses, we transformed the dataset by applying appropriate normalization strategies to each variable. The directionality of effect was made consistent so that higher values describing an endpoint represented a stronger biological effect. For example, with cell viability data, a stronger effect represents a decrease in the viability value versus untreated controls. Thus, to make the increase in values correspond to increases in the biological effect, all viability values were first normalized to untreated control means, which were considered 100% viable, and then the cytotoxicity values were formulated as a decrease in viability by subtracting the normalized viability values from 100%. Protective effects on the viability of hydrogen peroxide-treated cells were normalized to the mean values of two types of controls. The first, positive control, representing the baseline viability devoid of any protective effects in the presence of hydrogen peroxide was considered 0%. The second, negative control, consisted of cells treated with neither test substance, nor hydrogen peroxide. As such, they did not undergo any cell damage and were considered to represent the maximum reversal of oxidative damage or 100% protective effects. All experimental values were normalized as percentages according to their location in the interval between both controls. The hemolysis assay is based on measuring the absorbance of released hemoglobin. Sample values were again normalized to both the values with fully lysed erythrocytes, considered total hemolysis (100%) and spontaneous baseline hemolysis in untreated samples (0%). When baseline hemolysis was further reduced upon treatment due to membrane-stabilizing effects, values had a negative sign. With radical scavenging assays, the effects were compared and normalized to those of a reference antioxidant (Trolox), which was considered 100%. Among in silico data, the only values with more than one replicate were docking scores, representing the leading poses of the ligand in the predefined pocket of the enzyme (Supplementary Materials S1). They were inverted so that higher values represent higher affinity. Then, all variables with more than one replicate were aggregated by calculating the mean.

### 2.3. Correlation Analysis

The monotonic associations among potentially predictive in silico variables and in vitro outcomes, as well as between predictors and outcomes, were assessed by calculating Spearman’s rank correlation coefficients with the Hmisc (version 5.2-3) [63] R package. This exploratory approach was chosen due to the small observation size of the dataset because it is more robust to the impact of eventual outliers. This approach provides insight about the presence of highly correlated groups of variables. It is based on rank statistics and calculates pairwise correlation coefficients (ρ) and associated *p*-values. Cutoff thresholds for correlation (|ρ| > 0.6) and statistical significance (*p* < 0.05) were applied to isolate moderate-to-strong associations and control for type I errors. For symmetric correlation matrices (i.e., predictor–predictor and outcome–outcome comparisons), only variables demonstrating at least one significant correlation (beyond the predefined thresholds) with other variables were retained for visualization. For non-symmetric matrices (i.e., predictor–outcome comparisons), filtering was applied across rows and columns separately to ensure that each retained variable had at least one significant association in the corresponding dimension. Correlation plots were generated using the ggcorrplot (version 0.1.4.1) [64] R package.

### 2.4. Multiple Factor Analysis (MFA)

MFA is an eigenvector-based ordination method, which provides a framework to simultaneously analyze data, organized in groups of variables (blocks). Each group can contain either only quantitative or only qualitative variables and the sizes of groups can vary [65,66,67]. MFA is based on a combination of principal component analysis (PCA) for quantitative variables and multiple correspondence analysis (MCA) for qualitative variables. Some groups of variables (active) can be assigned to actively contribute to axis formation and determine the distances between variables/observations. Other groups of variables (supplementary) can be plotted only passively on the axes to show their strength of association with them. MFA first applies normalization inside each variable group to equalize groups’ contributions to the analysis. Then, PCA is performed individually on each block and the assigned weight to each group is the inverse of its first eigenvalue. Each normalized block is multiplied by its computed weight, creating a harmonized dataset where no single block dominates. Global factor axes that explain the variance across all blocks are then identified by performing PCA on the whole weighted matrix. Only then are supplementary groups plotted on the axes. Thus, mechanistic links across in vitro assays as active variables in the context of in silico predictions as supplementary variables can be identified. The reason for choosing only in vitro variables as active is that they are experimental results (and not predictions) and because of this forms a more reliable basis for determining relationships across multiple assays. We used FactoMineR (version 2.11) R package [68] for MFA and factoextra (version 1.0.7) [69] and ggplot2 (version 3.5.1) [70] R packages to generate plots.

### 2.5. Hierarchical Clustering on Principal Components (HCPC)

In order to identify natural groupings among compounds, we combined MFA with HCPC in FactoMiner (version 2.11) R package [68]. Before applying hierarchical clustering, missing values were first replaced with the respective group’s mean values. We chose the Manhattan distance metric for clustering as it is robust in high-dimensional spaces and less affected by outliers [71]. Automatic determination of the number of clusters was chosen as it finds the most statistically appropriate solution by analyzing the loss of inertia upon varying the number of clusters. Then, Ward’s method [72] was selected for hierarchical clustering as it minimizes the total within-cluster variance, creating compact and homogeneous clusters, which is particularly advantageous for small datasets. Finally, a consolidation step was performed to refine the results obtained from HCPC.

### 2.6. Exploratory Variable Selection for Key Predictor Identification

Three complementary methods were implemented to identify candidate in silico predictor variables. Each outcome variable was iteratively tested against all predictor variables with either standard lasso regression, robust lasso with the Huber loss function or random forest (RF)-based variable selection. Given the small sample size, the results were only intended to be interpreted as preliminary indicators of variable importance rather than definitive model outputs. Lasso regression was performed using the glmnet (version 4.1-8) [73,74] R package with an L1 penalty (α = 1) to induce sparsity by shrinking noninformative coefficients to zero. Leave-One-Out Cross-Validation (LOOCV) was employed to select the optimal regularization parameter (λ) and minimize overfitting. A robust variant of lasso was also implemented via the hqreg (version 1.4-1) [75] R package using the Huber loss function. LOOCV was similarly applied to determine the optimal λ. This method enhances the resilience of variable selection in the presence of extreme observations. Complementary to these regression-based approaches, RF variable selection was performed using a stepwise algorithm implemented in the steprf (version 1.0.2) [76] R package. With 1000 trees and 20-fold cross-validation, this ensemble method ranked variable importance via the KIAVI2 (Knowledge Informed Averaged Variable Importance 2) metric. Only predictors with stable importance scores were considered, acknowledging that the current dataset’s size may limit the generalizability of these rankings. The results from the three methods were used to derive an integrated consensus. Predictors identified by multiple methods were considered to have stronger preliminary evidence for relevance, while those selected by only one method were noted as tentative.

To enhance interpretability, the outcomes from the three methods for variable selection were integrated into an aggregated tabular summary using the kableExtra (version 1.4.0) [77] R package. Regression plots were then generated for outcome–predictor pairs that achieved unanimous selection across all methods. Each of the selected pairs was analyzed by fitting both an ordinary least squares (OLS) regression model and a robust regression model (using Huber’s loss [78]) to capture potential linear relationships while mitigating the influence of outliers. To allow for comparisons between the visualized slopes’ estimates, a jackknife resampling approach was implemented [79]. Namely, for each simple regression model, each observation was sequentially omitted, and the model was re-estimated on the remaining n − 1 cases. The slope estimates from these leave-one-out samples were used to compute a jackknife standard error (SE), which provided estimates of R^2^ and *p*-values for the slope coefficients.

## 3. Results

### 3.1. Data Preprocessing and Analysis

The analyzed dataset consists of multiple variables, characterizing compounds **5**–**5g** (see Supplementary Materials S2 and S3). The dataset is challenging to visualize and handle due to its size and heterogeneity. Variables were logically grouped (Table 1) to allow for summarized representation, analysis and visualization.

#### Correlations Among Predictor and Outcome Variables

**Figure 2:**
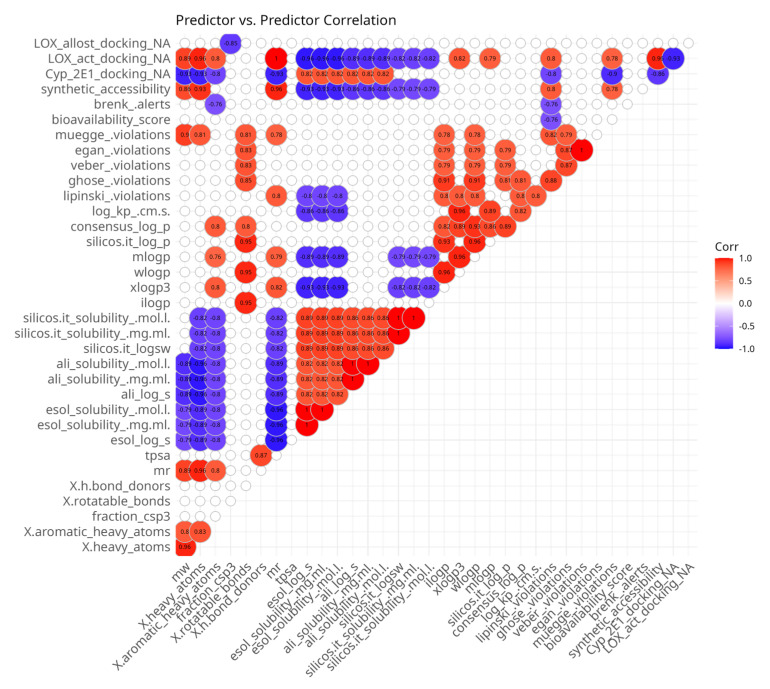
Spearman’s rank pairwise correlation map between predictor variables. A correlation cutoff (|ρ| > 0.6) and a significance threshold (*p* < 0.05) were applied to isolate moderate-to-strong associations. Variables without correlations over the cutoff were excluded from the plot. Red indicates a positive correlation and blue indicates an inverse correlation.

In order to demonstrate the relationships between predictor variables, measured for the compounds of the series **5**, **5a**–**5g**, a Spearman’s rank pairwise correlation map was generated. It was filtered to show only statistically significant moderate-to-strong correlations (Figure 2). The presence of multiple correlated predictor variables might hinder the interpretation of results, but randomly eliminating variables might lead to missing important causal relationships. The correlation map (Figure 2) shows that as expected, there are numerous associations among predictor variables. The most positively intercorrelated parameters are those related to water solubility. They are the outputs of three SwissADME [56] models, ESOL, Ali and SILICOS-IT, each of which is represented by three values: LogS, mg/mL and mol/L. ESOL and Ali methods rely on molecular descriptors and lipophilicity, while SILICOS-IT uses a fragmental approach corrected by molecular weight. All water solubility-related parameters are inversely correlated with some molecular mass-related parameters. ESOL and SILICOS-IT are also negatively associated with lipophilicity and synthetic accessibility.

Another group of intercorrelated parameters comprises lipophilicity-related paramters which predict the partition coefficient between n-octanol and water (logP o/w). They do not measure solubility in water or lipids directly; instead, they provide insights into how lipophilic a compound is, which can indirectly influence its solubility in aqueous environments. The consensus LogP is positively associated with all lipophilicity-related parameters because it is the arithmetic mean of the values predicted by all of the methods. iLOGP, WLOGP and SILICOS-IT LogP are positively intercorrelated. MLOGP and XLOGP3 are also highly intercorrelated. However, there are no significant correlations between both groups of lipophilicity-related parameters. iLOGP, WLOGP, MLOGP and consensus LogP are positively correlated with the number of rotatable bonds.

Molecular size-related parameters are also positively intercorrelated. Molecular weight (mw), molar refractivity (mr), number of heavy atoms (x.heavy atoms) and number of aromatic heavy atoms (x.aromatic heavy atoms) are all related to the number of atoms and the volume of the molecule. Most of them are used in some of the water solubility models and are inversely correlated with them, with the exception of the lack of association between the SILICOS-IT-related parameters and mw. These parameters are positively correlated with the predicted synthetic accessibility.

The ADME-related parameters represent membrane permeability, pharmacokinetic properties and potential for oral bioavailability. Log Kp, bioavailability score, and Lipinski, Veber, Egan and Muegge violations highlight different aspects of compounds. The eponymous systems of violations point to the presence of properties, which decreases the likelihood of good oral bioavailability. Log Kp represents the permeability coefficient of a compound across biological membranes, measured in centimeters per second (cm/s), and the bioavailability score is a composite metric that estimates the likelihood of oral bioavailability. There are some correlations (positive or negative) between those parameters. Interestingly, within the current dataset, the skin permeability coefficient Log Kp is positively correlated with the number of Lipinski’s rule violations. As expected, the number of Lipinski’s rule violations are positively correlated with Ghose and Muege violations, which build upon it by adding more rules and are negatively correlated with the bioavailability score. The best correlation observed is between Veber and Egan violations. Ghose violations are positively associated with all Muege, Egan and Veber violations. Lipinski violations are positively correlated with some lipophilicity parameters and molar refractivity and are negatively associated with some water solubility parameters. Veber, Egan and Muegge violations are positively correlated with some water solubility parameters and the number of rotatable bonds. Muegge violations are also positively correlated with molar refractivity, mw and the number of heavy atoms.

Among the docking parameters, 5-LOX active site affinity was positively correlated with molecular size and some lipophilicity-related parameters and negatively associated with water solubility parameters and docking affinity to Cyp 450 2E1. Inversely, Cyp 450 2E1 docking affinity was positively correlated with water solubility and inversely correlated with molecule size. 5-LOX allosteric site docking affinity was only negatively correlated with the fraction of carbon atoms that are in the sp^3^ hybridization state.

The plot also allowed for the identification of several predictor variables which are less intercorrelated with others: sp^3^-hybridized carbon atoms, the number of H-bond donors, TPSA and Brenk alerts.

**Figure 3:**
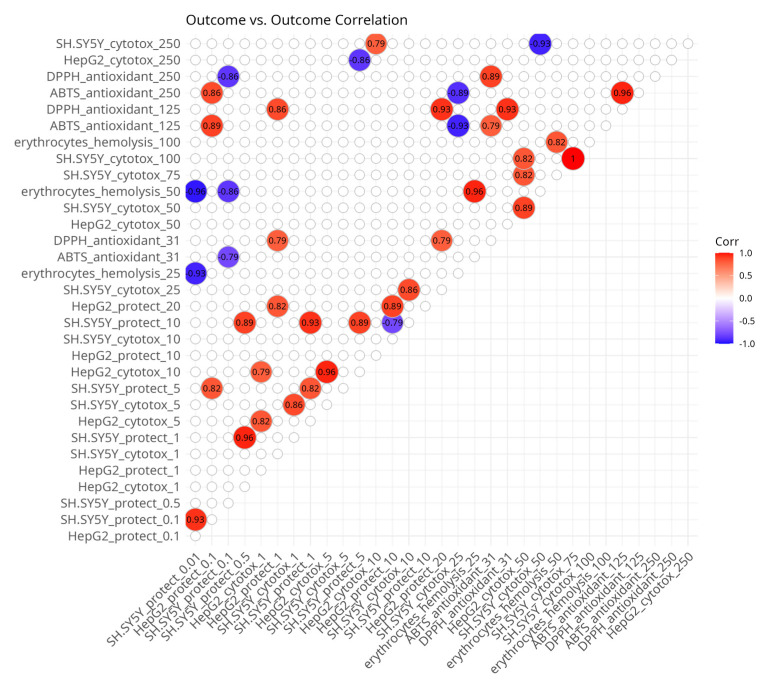
Spearman’s rank pairwise correlation map between outcome variables. A correlation cutoff (|ρ| > 0.6) and a significance threshold (*p* < 0.05) were applied to isolate moderate-to-strong associations. Variables without correlations over the cutoff were excluded from the plot. Red indicates a positive correlation and blue indicates an inverse correlation.

Among the outcome variables (Figure 3), correlations were also anticipated because some variables represent treatments with the same compounds on the same model systems, but at different doses. The observation of some positive and negative correlations among outcome variables might help explain some biological associations. There were positive associations between the different concentrations of the same experiment types with the exception of the negative correlation between cytotoxicity in SH-SY5Y neuroblastoma cells, which showed that the same substances which caused the strongest effects at 50 µM had the lowest effects at 250 µM and vice versa.

There were positive correlations between different concentrations of the ABTS and DPPH radical scavenging assays. The cytotoxicity values of HepG2 cells at 10 µM and 50 µM and of SH-SY5Y cells at 250 µM and 25 µM, respectively, were also positively associated. There was a positive correlation between the protective effects against hydrogen peroxide-induced damage in SH-SY5Y cells at 5 µM and the protective effects against hydrogen peroxide-induced damage in HepG2 cells at 0.1 µM. Protective effects in HepG2 cells at 0.1 µM were correlated with ABTS assay outcomes at 125 µM. Protective effects in HepG2 cells at 1 µM and 20 µM were associated with the DPPH assay at 31 µM and 125 µM.

The negative correlations were mostly seen with the protective effects in SH-SY5Y cells. They were observed at 10 µM with protection in HepG2 cells also at 10 µM, at 5 µM with HepG2 cytotoxicity at 250 µM, at 0.1 µM and 0.01 µM with hemolysis at 50 µM, and at 0.01 µM with ABTS at 31 µM and DPPH at 250 µM.

**Figure 4:**
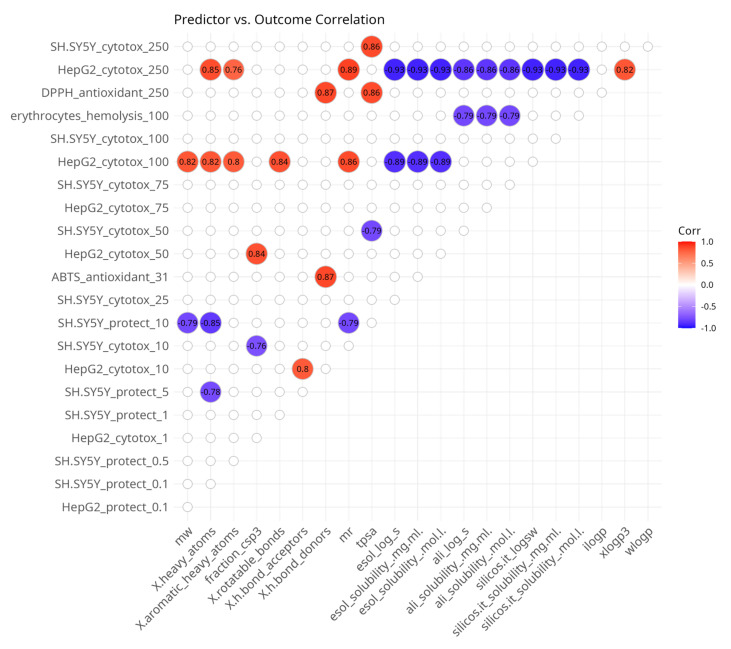
Spearman’s rank pairwise correlation map between predictor and outcome variables. A correlation cutoff (|ρ| > 0.6) and a significance threshold (*p* < 0.05) were applied to isolate moderate-to-strong associations. Variables without correlations over the cutoff were excluded from the plot. Red indicates a positive correlation and blue indicates an inverse correlation.

The correlation map presented in Figure 4 is asymmetric and shows the presence of significant associations between in silico predictor variables and in vitro outcome variables. Most of the correlations were observed with the cytotoxicity experiments on HepG2 cells and only some of them with SH-SY5Y cells. Cytotoxicity to HepG2 cells at 100 µM and 250 µM was positively associated with molecular size-related predictors and the lipophilicity parameter XLOGP3 and negatively associated with water solubility parameters. At concentrations of 10 and 50 µM, HepG2 cytotoxicity was positively correlated with the number of H-bond acceptors and the fraction of sp3-hybridized carbon atoms, respectively.

Cytotoxicity to SH-SY5Y cells was positively associated with TPSA at 50 µM and negatively correlated with TPSA at 250 µM. At 10 µM, it was inversely related to the fraction of sp3-hybridized carbon atoms.

A few correlations were observed with in vitro data from assays for oxidative stress protection. Both correlations were with SH-SY5Y cells. At 5 and 10 µM, the protective effects were negatively correlated with molecular size-related parameters. Hemolysis at 100 µM was inversely correlated with water solubility-related predictors and DPPH at 250 µM was positively associated with the number of H-bond donors and TPSA.

### 3.2. Multiple Factor Analysis

The presence of highly intercorrelated variables is a reason to use exploratory approaches handling groups. MFA is such a method and we applied it to explore any inherent patterns in the dataset. In vitro variables were used as active and in silico variables were supplementary to provide context about possible causative relationships.

#### 3.2.1. Eigenvalues of Principle Components (PCs)

**Figure 5:**
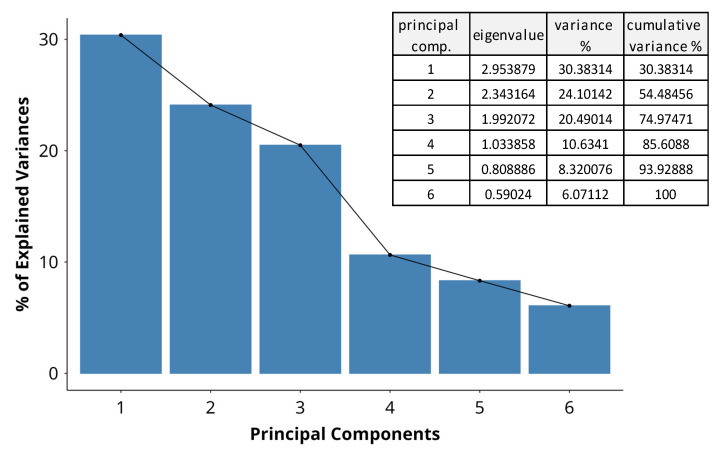
A scree plot indicating the percentage of variance in the dataset, explained by each principal component. The bars represent the variance by dimension, and the adjacent table indicates the eigenvalues and cumulative variance across dimensions.

Differences within the data were aggregated and represented as principal component (PC) variables by MFA. The scree plot (Figure 5) summarizes the variance in the dataset explained by each PC. PC 1 and PC 2 are the most significant, explaining more than half of the total variance, at 30% and 24%, respectively. PC 3 and PC 4 have eigenvalues over 1 and explain a further 20% and 11%, bringing the cumulative variance across the first four components to 85%. The sharp decline in explained variance at PC 4 suggests that the majority of the variability in the data can be captured with the first three dimensions. PC 5 and PC 6 explain only 8% and 6%, contributing only marginally to the total variance. They are unlikely to substantially contribute to the interpretation of major trends in the dataset. By applying the Kaiser criterion for further analysis, we disregard PCs 5 and 6, which have eigenvalues lower than 1 (Figure 5).

#### 3.2.2. Group Variable Contribution to PC Dimensions

**Figure 6:**
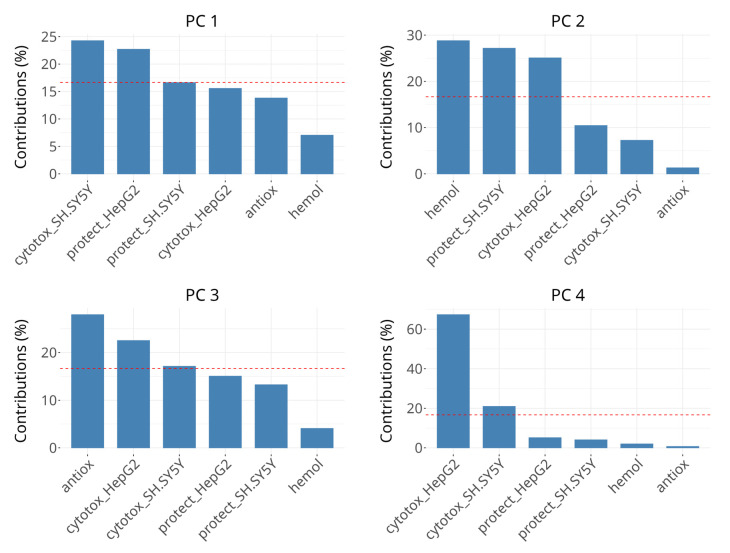
Scree plots showing the percentage of explained variance for PCs 1 to 4. The bars represent the variance explained by groups in individual dimensions. The horizontal reference dashed line corresponds to the expected value if the contribution was uniform.

The contributions of the groups of variables to the first three dimensions of the analysis are presented in Figure 6. Distinct groups influence the dimensions disproportionately, suggesting that each dimension captures unique aspects, resulting from specific variables dominating the variance structure.

The variability in PC 1 is dominated by the groups describing cytotoxicity effects on SH-SY5Y (~25%) and viability protection on HepG2 (~24%), followed by viability protection on SH-SY5Y and cytotoxicity in HepG2, the latter two contributing ~15–20% each, which is below the expected value if the contributions were uniform. The rest of the groups contribute minimally (<10%). The interpretation of PC1 seems to be related to cellular processes and responses in oxidative stress conditions, but also affects cell viability when there is no oxidative damage.

The most significant contributors to the variability in PC 2 are hemolysis of erythrocytes, viability protection on SH-SY5Y and cytotoxicity in HepG2, each explaining about 25–30%. Other variables have negligible contributions in this dimension. Its interpretation may be related to cell membrane-related events, which cannot be attributed only to cell death or only to survival in oxidative stress conditions. The presence of viability protection on SH-SY5Y and cytotoxicity in HepG2 in both PC 1 and PC 2 could be due to concentration-dependent differences in subgroups, for example the negative correlation between cytotoxicity in SH-SY5Y cells after 50 µM and 250 µM treatments or due to complex multitarget interactions.

In PC 3, radical scavenging is the dominant contributor (>25%), followed by cytotoxicity in HepG2 and borderline cytotoxicity in SH-SY5Y (18–23%). Changes in the coordinates in PC 3 can be interpreted as primarily related to direct radical scavenging activity. PC 4 is strongly dominated by cytotoxicity in HepG2, which accounts for over 60% of the variance, followed by cytotoxicity in SH-SY5Y with over 20%. It might be interpreted as being influenced by factors driving cell death predominantly in HepG2 cells and, to much lower extent, in SH-SY5Y cells.

#### 3.2.3. Principle Component Plot

**Figure 7:**
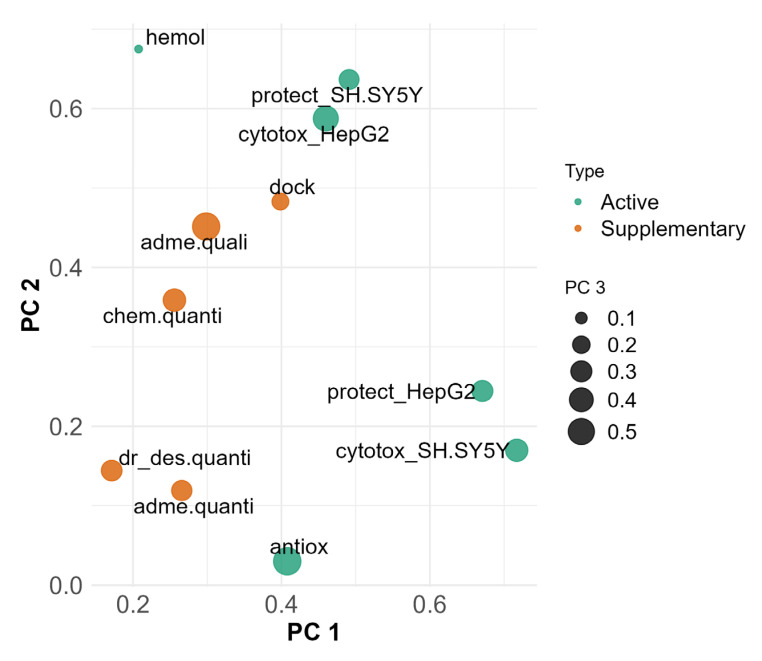
MFA projection plot on PC 1 (x axis), PC 2 (y axis) and PC 3 (dot size), highlighting relationships between active and supplementary groups. Active variable groups (green) participate in formation of PCs. Supplementary variable groups (orange) are used for interpretation purposes and do not participate in formation of PCs.

For the identification of patterns shared between groups, we plotted them on the primary three PC dimensions (Figure 7). In this plot, the active variables (green dots) hemolysis, cytotoxicity in HepG2 and viability protection on SH-SY5Y are grouped in the upper middle part. In the lower right part of the plot, a distinct cluster forms between cytotoxicity in SH-SY5Y, viability protection in HepG2 and radical scavenging. The size of the dots represents PC3 or, as shown before, associations with radical scavenging, and shows clustering of active variables despite the weaker contribution to explaining the variance in PC 3 compared to PC 1 and PC 2. Viability protection on both HepG2 and SH-SY5Y and cytotoxicity in SH-SY5Y have similar dot sizes, indicating close PC3 coordinates. Cytotoxicity in HepG2 is separate from the rest of the active variables in PC 3, which is also observed with hemolysis.

Supplementary groups of variables (Figure 7, orange dots) not participating in the formation of the principal components were added for the purpose of contextual interpretation. Their presence in the plot allows for identification of correlations and proximity to groups of active variables, which could indicate shared underlying mechanisms.

Docking affinities, ADME and solubility qualitative predictions and chemical property predictions are grouped together. They are closest and most related to cytotoxicity in HepG2, viability protection on SH-SY5Y and hemolysis.

In PC 3, most of the active and supplementary variables are moderately represented. ADME and solubility quantitative predictions, chemical property predictions and drug-likeness are grouped together and with the active variables, except for hemolysis, antioxidant assays and cytotoxicity in HepG2.

#### 3.2.4. Individual Variable Contribution to PC Dimensions

**Figure 8:**
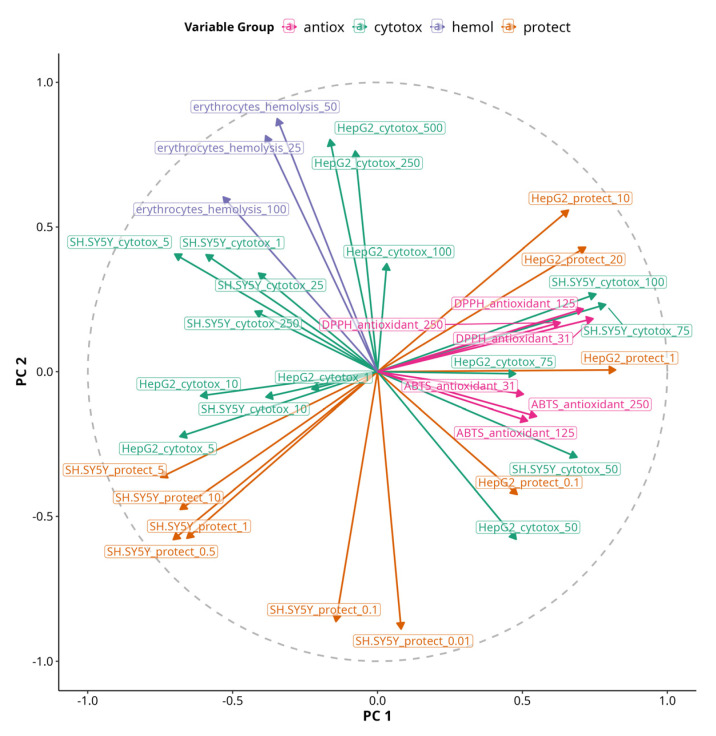
An MFA correlation circle plot of PC 1 and PC 2. The length of vectors projecting from the center to the circle represent the quality of continuous variables. The angle of lines and their orientation towards or against PC1 or PC2 axes shows their relationship to them. Variables are color-coded based on their group origin.

To bridge our understanding about the directionality of correlations between single variables and their contribution to MFA PC dimensions, we projected a correlation circle onto PC 1 versus PC 2, which together explain 54% of the variability in our data (Figure 8). Variables’ arrows pointing in the same direction along PC dimensions reflect shared or synergistic contributions to the variability in the dataset and vice versa.

The variable most aligned with PC 1 was HepG2 viability protection under 1 µM treatment, while the most aligned with PC 2 was HepG2 cytotoxicity under 250 and 500 µM treatments. Antioxidant assays were also positively correlated with PC 1, but DPPH had slightly positive coordinates on PC 2, while ABTS had slightly negative coordinates. Inversely related to PC 2 was viability protection of SH-SY5Y under 0.01 and 0.5 µM treatments. Hemolysis was correlated with PC 2 and slightly inversely correlated with PC 1. However, the most pronounced effects on erythrocytes were negative as no significant hemolysis was observed with any of the treatments, but some of them caused membrane stabilization and had negative values. Thus, the arrow essentially shows the opposite of the presence of membrane-stabilizing effects, so we can hypothesize that the protective effects on SH-SY5Y at 0.01 and 0.5 µM are associated with membrane stabilization. Viability protection on SH-SY5Y under 0.5 µM treatments was inversely correlated with both PC 1 and PC 2. Viability protection on HepG2 under 10 and 20 µM treatments was positively correlated with both PC 1 and PC 2, indicating the presence of both protective and cytotoxic effects with increasing treatment concentrations.

#### 3.2.5. Partial Individual Coordinates

**Figure 9:**
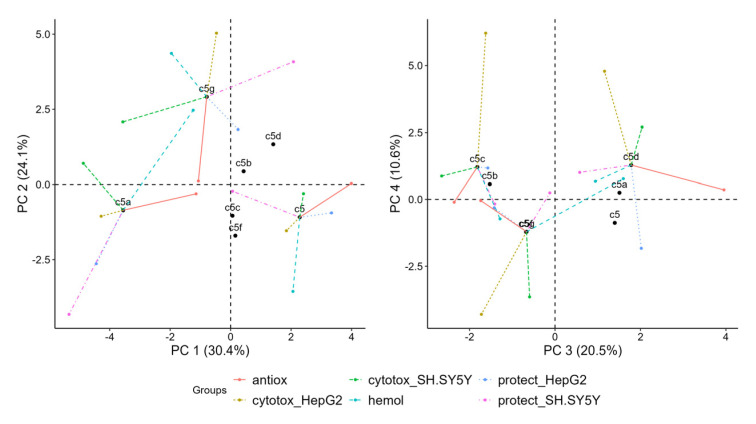
Projection of individual compounds and their partial points onto first four principal components derived from multiple factor analysis (MFA).

The projection of individual compounds and their partial points on the first four PCs is shown in Figure 9. They reveal which are the variables that draw observations (compounds **5**, **5a**–**5g**) together or apart on the PC plot.

In the left plot in Figure 9, **5g** has relatively strong positive coordinates on PC 2, **5** has positive coordinates on PC 1 and **5a** has negative coordinates on PC 1. The partial points show that the variables which separate **5** from other points in dimensions 1 and 2 are hemolysis, which is negative on PC 2, and radical scavenging, which is positive on PC 1. **5g** is separate predominantly because of its positive coordinates in PC 2 due to cytotoxicity in HepG2 and hemolysis. **5a** is separated because of the prominently negative coordinates of all partial points except for hemolysis and radical scavenging. It also has negative coordinates on PC 2 due to viability protection on both SH-SY5Y and HepG2.

In the plot of PC 3 and PC 4 (Figure 9), **5c** and **5d** are highest on PC 4 mostly due to cytotoxicity in HepG2, but they are on the opposite ends on PC 3 due to the positive coordinates of **5d** regarding radical scavenging and the negative coordinates of **5c** due to both cytotoxicity in SH-SY5Y and radical scavenging. **5g** is negative regarding cytotoxicity in HepG2 and SH-SY5Y on both PC 3 and PC 4.

### 3.3. Hierarchical Clustering of Compounds

**Figure 10:**
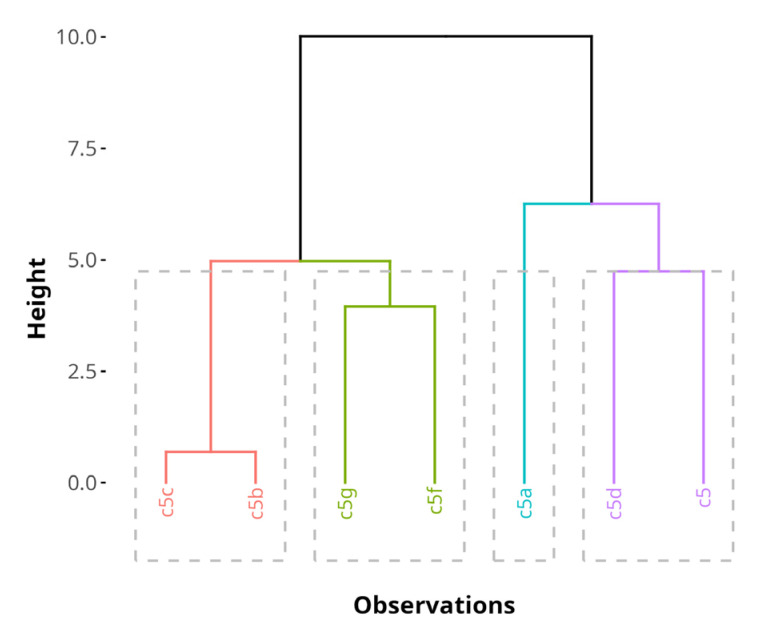
A hierarchical cluster tree, generated by HCPC on the MFA results before cluster tree consolidation. The clusters are color-coded. The height of the horizontal lines represents similarity between compounds.

Hierarchical clustering of compounds based on the PC dimensions from the MFA reveals four distinct biological profiles, as shown in Figure 10. The dendrogram represents the proximity of compounds in the MFA space before the cluster consolidation step. The consolidation step was added to refine the initial hierarchical clustering solution by reassigning observations to clusters in a way that minimizes within-cluster variability, which results in more robust and interpretable clustering. Consolidation resulted in reallocation of **5f** to the same cluster as **5b** and **5c**.

Table 2 shows a summary of the variables driving clustering and their characteristics after consolidation. PC 1 and PC 3 explained nearly all of the variability which drove cluster formation. Eta^2^ values were 0.98 for PC 1 and 0.94 for PC 3. High cluster eta^2^ values show that the differences between clusters account for most of the variation observed in the MFA PCs of the dataset. The v-test in the clustering results represents how strongly a variable contributes to distinguishing a given cluster. A large absolute value of the v-test (>2) indicates that the variable is significantly over- or underrepresented in that cluster. The associated *p*-value shows the likelihood of type I errors with the v-test statistic values. Cluster 1 had significantly lower mean values on PC 1 (v-test  =  −2.07, *p*  =  0.039) and cluster 3 had significantly lower mean values on PC 3 (v-test  =  −2.01, *p*  =  0.044). This demonstrates the robustness of the clustering and suggests that the identified subgroups differ meaningfully based on the MFA outputs.

Cluster 1 consists of **5a**. It is significantly enriched in quantitative variables related to SH-SY5Y viability protection after treatment with 1 µM, 5 µM and 10 µM.

Cluster 2 includes **5g**. While no significant contributions from principal dimensions are observed, the cluster is significantly enriched in quantitative variables such as marginal increases in the docking affinities to MPO and the allosteric center of 5-LOX with one unit each, as well as a very subtle 2% increase in HepH2 cytotoxicity at 500 µM.

Cluster 3 comprises **5b**, **5c** and **5f**. It is characterized by the presence of only one H-bond donor, compared to the average of 1.57 across all data (v-test = −2.45, *p* = 0.014).

Cluster 4 includes **5** and **5d**. This cluster is significantly enriched in variables related to radical scavenging activity, such as DPPH and ABTS radical scavenging at all treatment concentrations, improved viability protection on HepG2 cells at 0.1 and 1 µM and a lower Log Kp, indicating a preference for water phases and a lower propensity to diffuse through membranes. Cluster 4 is primarily characterized by compounds with strong radical scavenging properties, aligning with the functional relevance of the studied antioxidant activity.

### 3.4. Predictor Variable Choice

After observing some correlations and natural groupings between in vitro and in silico variables, we implemented three different variable selection methods to identify the most important in silico predictors of the in vitro results. Predictions were of inherently limited validity due to the small number of observations in the dataset. For this reason, we applied a consensus approach to compensate for the weaknesses of single models. This way, in silico predictors disregarded by the previous analyses could also be identified. The three feature selection methods showed considerable variability in which predictors they selected (Supplementary Materials, Table S4). Out of 177 predictor–outcome combinations, the majority (115) were selected by only one of the three methods. Another 52 instances were selected by two of the methods, and only 10 predictor–outcome combinations (~6%) were commonly selected by all three methods. Notably, the lasso and robust methods had the greatest overlap, jointly selecting 35 instances (~20%) without RF, whereas robust and RF without lasso overlapped on 28 instances (~16%), and lasso and RF without robust overlapped on 19 instances (~11%). This suggests that the lasso and robust methods tend to select similar features more often and RF is closer to the robust model compared to lasso. Each model also had a set of unique picks. RF selected 76 instances that neither lasso nor Huber chose, Huber had 31 unique instances, and lasso had only 8 unique instances. Thus, the RF model tended to include many features that the linear models did not, whereas lasso largely intersected with the robust regression’s choices.

**Figure 11:**
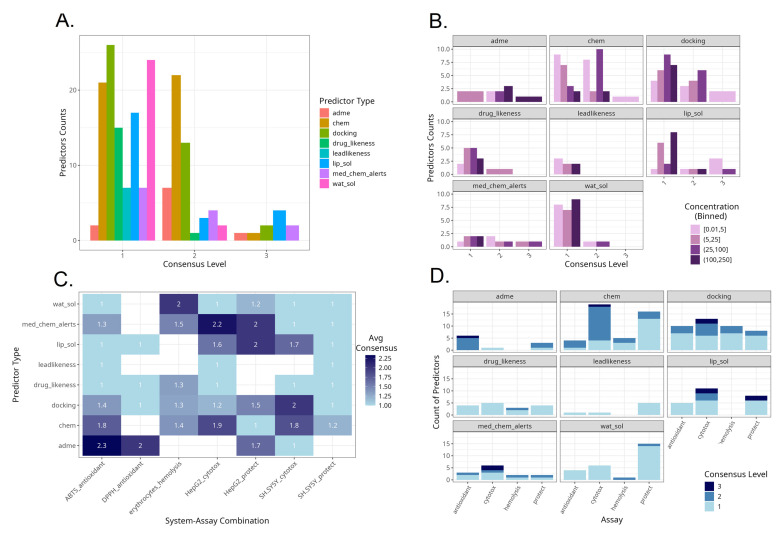
Summaries of model consensus levels across predictors and assays. (**A**). Counts of predictor types by consensus level. (**B**). Counts of predictor types by consensus level and in vitro assay concentration range. (**C**). Average consensus levels by predictor type and specific experimental system. (**D**). Counts of predictor types by consensus level and in vitro assay type.

A visual summary of the consensus between the output from the three models and how it varies by predictor, model, treatment concentration and assay is presented in Figure 11. Most of the instances with full consensus among models were related to the in silico predictors of lipophilicity, docking and medicinal chemistry alerts (Figure 11A). No water solubility-, lead-likeness- and drug-likeness-related predictors were selected with full consensus. Molecular size-, water solubility- and docking-related parameters were the most selected by a consensus of two methods.

Across predictors, there were different consensus levels according to the in vitro treatment concentrations applied (Figure 11B). With ADME-related and docking-related parameters, the highest consensus levels were obtained at higher treatment concentration levels. In contrast, with molecular size-related parameters, the highest consensus level was achieved at lower and medium concentrations. With medical chemistry-related filters, the highest consensus levels were achieved at the intermediate concentrations between 5 and 100 µM.

Some experimental types were characterized with high average consensus levels (Figure 11C). These were ADME-related predictors for the outcomes of the ABTS assay, followed by medicinal chemistry-related filters as predictors for HepG2 cell line cytotoxicity, and then ADME-related parameters for predicting the DPPH assay, water solubility for hemolysis, both medical chemistry filters and lipophilicity for protective effects on HepG2 cells and docking for cytotoxicity in SH-SY5Y. Among the selected in silico–in vitro variable pairs, those containing protective effects against oxidative damage in SH-SY5Y cells were the least predictable, indicated by the lowest average consensus scores. This implies that the protective effect outcome in SH-SY5Y is more complex or noisy. Each model selected different predictors with little agreement, perhaps reflecting multiple mechanisms or a lack of a single dominant driver. Hemolysis had moderate average scores, albeit consistent across all types of predictors.

The predominance of consensus levels across predictors and assay types is presented in Figure 11D. ADME-related predictors were selected with high consensus mostly for the radical scavenging assays. Molecular size-related parameters as well as docking studies were selected with high consensus scores mostly for cytotoxicity-based assays.

**Figure 12:**
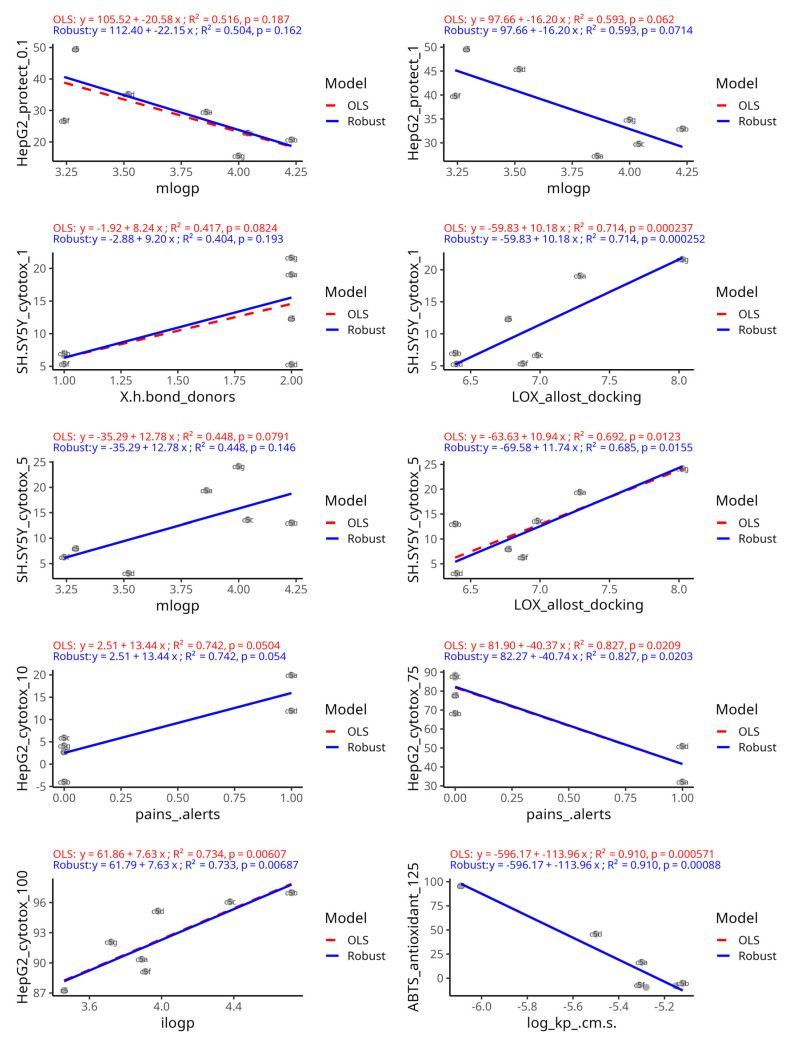
The outcome–predictor plots identified unanimously by the three variable selection methods. Potential linear relationships were demonstrated by fitting both an ordinary least squares regression model (red dashed line) and a robust regression model using Huber’s loss (blue line). For comparison slopes, R^2^ and slope *p*-values were generated by using a jackknife resampling approach.

We further examined the set of predictor–outcome combinations that all three models unanimously selected (Figure 12). The regression plots demonstrate the strength and direction of each association and how robust they are to outliers or influential points. The robust regression with the Huber loss function in all plots was in close agreement with the OLS regression. This implies that none of the consensus predictor relationships were driven by outlier artifacts and that they hold true for most compounds. The high R^2^-values and low *p*-values confirm that the outcomes are stable and indicate statistical robustness even when individual data points are iteratively removed by using the LOOCV approach. Furthermore, the fact that RF, which unlike the other two methods is a nonlinear, tree-based model, also identified the same predictor–outcome relationships reinforces the notion of the predictive importance of the selected variable pairs. Namely, the strongest regression with the best fit was observed with the outcome ABTS under 125 µM treatment versus the permeability coefficient Log Kp, which were inversely associated. The positive association between cytotoxicity in HepG2 cells at high concentrations and lipophilicity was also among the strongest observed. SH-SY5Y neuroblastoma cytotoxicity at low concentrations was positively related with lipophilicity parameters and LOX allosteric docking affinity. The protective effects on HepG2 cells at low concentrations were inversely associated with lipophilicity probably due to the presence of low-grade cytotoxicity. Among the consensus-chosen variables, weaker associations were observed with SH-SY5Y cytotoxicity at low concentrations, predicted by lipophilicity, and with HepG2 cytotoxicity, predicted by PAINS alerts, indicated by a positive association at 10 µM and an inverse association at 75 µM. Some slopes were very slightly attenuated with the robust regression, compared to OLS, which was observed only with the pairs mLogP—hepatoprotective effects at 0.1 µM, the number of H-bond donors—neurotoxicity at 1 µM, and 5-LOX allosteric site docking affinity—neurotoxicity at 5 µM. This was an indication that one or two points, perhaps representing compounds with extremely low scores, had some disproportionate influence on the slope.

## 4. Discussion

The results from the exploratory analysis of in vitro and in silico data, characterizing the effects of the series of hydrazide-hydrazone compounds **5**–**5g**, showed the presence of some distinct profiles among them. Compounds **5b**, **5c** and **5f** clustered together and were characterized by higher variability in the protective effects on SH-SY5Y cells and less H-bond donors. Compounds **5** and **5d** were the best radical scavengers and in vitro hepatoprotectors. They were also characterized by a lower membrane permeability constant. Compound **5a** was the most pronounced in vitro neuroprotector, but a weaker hepatoprotector. Compound **5g** was characterized by the best docking affinities to MPO and at the allosteric site of 5-LOX.

**Figure 13:**
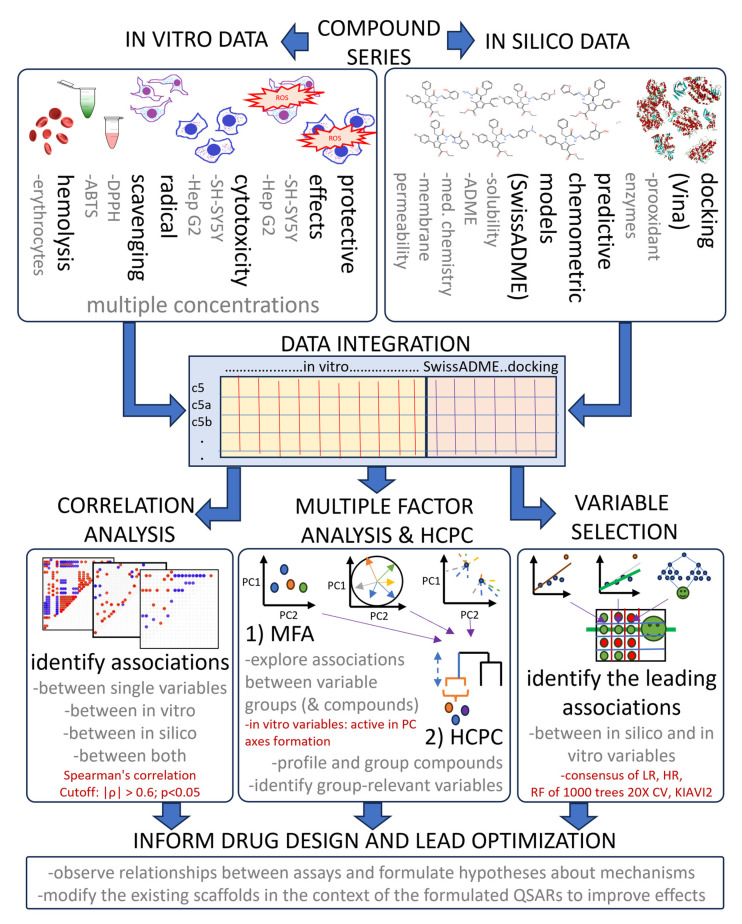
A flowchart of the used exploratory data analysis approach, highlighting data integration, analysis and interpretation steps. Text in red shows important parameters. |ρ|—absolute value of correlation, LR—linear regression, HR—robust regression using Huber’s loss, RF—random forest, CV—cross-validation.

Despite the widespread adoption of multivariate analytics, integrated workflows that simultaneously analyze in vitro responses at different concentrations with in silico descriptors, using MFA with HCPC, remain surprisingly rare. Our approach implements three comprehensive tiers aimed at identifying associations between variables driving the differences between compounds. The relationships identified by MFA are based on experimental data, while predictions are only used for their interpretation (Figure 13).

A report by Mkhayar et al. (2003) [80] describes the investigation of non-small-cell lung cancer agents and the use of PCA for variable selection, followed by clustering, QSAR modeling and molecular docking of the top hits. However, this tiered strategy does not include concurrent evaluation of all predictors, which in our case is done by MFA. Thus, it does not fully utilize the opportunities for discovering latent relationships among bioassay endpoints and computational parameters.

In our study, MFA showed that cytotoxicity to HepG2 cells is correlated with qualitative ADME and solubility predictions. Protection on hydrogen peroxide-damaged HepG2 cells is associated with radical scavenging. Hemolysis and radical scavenging are associated with ADME and solubility quantitative predictions. Docking is correlated with cytotoxicity in both SH-SY5Y and HepG2 cells.

A consensus approach combining three complementary variable selection methods was implemented to identify the strongest causal relationships in the context of multiple intercorrelated in silico predictors. Given the limited sample size, the aim of this analysis was solely hypothesis generation and variable prioritization rather than robust predictive model training and use. This approach filtered out the majority of unstable predictors and reinforced the few truly significant ones. For instance, lipophilicity, membrane permeability and PAINS were evident in all methods, instilling confidence that they are not just model-specific artifacts. Model agreement was highest for cytotoxicity endpoints, moderate for biochemical antioxidant and hemolysis assays, and low for the protective assays in neuron-derived cells. This highlights the possibility for modulating some of them by drug design based on the observed relationships.

A key finding across our analyses is the outsized role of lipophilicity in determining both efficacy and toxicity outcomes. This is expected as the role of lipophilicity in cytotoxicity is a foundational biological concept. LogP-related descriptors emerged as top predictors across all exploratory methods. In both HepG2 and SH-SY5Y cell lines, more lipophilic compounds showed higher cytotoxicity. This can be explained by the fact that highly hydrophobic molecules can accumulate in cell membranes and organelles, disrupting their function or triggering cell death pathways. Conversely, moderately lipophilic or polar compounds may be less likely to cause such damage. An optimal lipophilicity range is crucial for candidate drugs to maintain the balance between efficacy and safety. Our results align with the “3/75 rule” in medicinal chemistry, which states that compounds with ClogP lower than 3 and TPSA higher than 75 are less likely to cause toxicity at plasma concentrations below 10 μM. However, this rule does not seem to help a drug to progress through clinical trials [81].

Measures of molecular polarity are inversely related to toxicity and lipophilicity. In the current dataset, the only parameter with which TPSA was positively correlated was the number of H-bond donors. The H-bond donor and acceptor ratio has been shown to impact permeability and water solubility [82]. Their disbalance results in frustrated solvation and secondary electrostatic interactions. Hydrogen-bond donors seem to be more problematic in drug design than acceptors as most of a drug’s polarity is typically due to acceptors, while donors can decrease solubility, membrane permeability and bioavailability. Despite this, the most neuroprotective compound in the series **5a** contains an extra hydrogen-bond-donating group in its structure relative to some others, which may partly contribute to its favorable activity/toxicity balance.

Future optimized analogs of hydrazide-hydrazones might be tuned for slightly higher H-bond acceptor counts to reduce cytotoxicity while still maintaining enough brain penetrance for neuroprotective action.

ADME-related descriptors like Log Kp, which reflect the ability of a compound to permeate barriers, were shown to correlate with antioxidant activity in cell-free assays. In the ABTS assay, lower permeability, which usually corresponds to higher water solubility and polarity, exhibited better antioxidant performance. When assessing antioxidant compounds in vitro, one should consider physicochemical properties because artefacts might arise if a very hydrophobic substance precipitates or does not fully react. Thus, a moderately polar compound might perform better in an in vitro assay.

The pan-assay interference compounds (PAINS) alert was another significant predictor identified across all stages of the analysis, particularly for cytotoxicity in HepG2 cells. Our data showed that compounds flagged with one or more PAINS substructures tended to exhibit higher hepatotoxicity at 10 µM, but lower hepatotoxicity at 75 µM. PAINS are defined by structural motifs that are notorious for causing false signals in assays through non-specific reactivity, for example, through colloidal aggregation, redox cycling, covalent modification of proteins or fluorescence interference [83]. PAINS have been catalogued extensively specifically because they recur in many “false hit” compounds. For example, catechol or hydroquinone moieties which undergo redox cycling are classic PAINS, but can also be important components of pharmacophores and toxicophores [84,85,86]. A possible explanation for PAINS alerts leading to higher effects at lower concentrations, but not at higher concentrations, might be assay interference, which is negligible compared to stronger observed effects at higher concentrations. Moreover, pharmacological effects might manifest at concentrations higher than those causing assay interference. From a practical standpoint, the results for compounds triggering PAINS alerts should be treated with caution as they are more likely to elicit confounding biological effects. Thus, the presence of PAINS alerts should be taken into consideration as it adds significant unpredictability to the expected biological effects.

The in silico docking scores for pro-oxidant enzymes offer clues about possible mechanisms of action. We docked the compounds against enzymes MPO, 5-LOX, NOX, COX-2 and CYP2E1. They have been known to be involved in oxidative stress pathways and have been recognized as potential pharmacological targets. Benzoic acid hydrazides have been shown to inhibit MPO [87]. There are reports that pyrazole-linked hydrazones can inhibit 5-LOX, comparable or superior to the clinical 5-LOX inhibitor zileuton [88]. This shows that hydrazone-containing compounds have the potential for interaction with 5-LOX activity. Our correlation analysis (Figure 2) among all in silico predictors showed that with molecules in the current dataset, the higher fraction of sp3-hybridized atoms may be associated with lower docking scores at the allosteric binding site of 5-LOX. This could be due to increased steric hindrance, altered lipophilicity profiles or reduced molecular flexibility [89]. 5-LOX inhibition is generally considered anti-inflammatory and potentially neuroprotective [90]. Many known 5-LOX inhibitors also possess antioxidant or radical scavenging activity [91]. Compounds that bind 5-LOX tightly could share a certain lipophilicity or reactivity that inherently makes them more toxic to cells. 5-LOX has a dual action—leukotriene B4 synthesis by epoxide hydrolase activity and aminopeptidase activity to cleave the N-terminal proline of a pro-inflammatory tripeptide, prolyl-glycyl-proline [92]. Pyrimidine analogues can both inhibit 5-LOX and be moderately antioxidant in DPPH assays [93]. Enzymes with similar catalytic or allosteric sites to 5-LOX may be inadvertently targeted by 5-LOX inhibitors and result in cytotoxicity in in vitro conditions [94]. In in vitro studies, 5-LOX and COX-2 inhibitors showed protective effects against trimethyltin-induced oxidative stress in SH-SY5Y cells [95], despite 5-LOX mRNAs not being detected in SH-SYY cells [96]. It has been shown that 5-LOX inhibitors can also cause cytotoxicity in cells which do not express 5-LOX. For example, the non-redox-type inhibitor Rev-5901 causes cytotoxicity in Panc-1, HeLa and U937 cells, which were shown not to express 5-LOX under the respective culture conditions. The effects were even stronger compared to their morphologically related 5-LOX-positive counterpart cell lines Capan-2 and THP-1. This demonstrates the presence of mechanisms related to off-target effects [97]. It is possible that Rev-5901 is not only a 5-LOX inhibitor but also an antagonist of the cysteinyl-leukotriene receptor [98]. The allosteric 5-LOX inhibitor 3-O-acetyl-11-keto-β-boswellic acid (AKBA) can also cause a dose-dependent surge in ROS in MCF-7 cells with low 5-LOX expression levels [99].

The depmap portal online tool [100,101] revealed that across HepG2 and SH-SY5Y cell models, 5-LOX and COX2 encoding genes had negligible or absent mean expression values. NOX1 exhibited absolute expression values remaining low in both cell types. Batch-corrected log2-transformed TPM values for these cell lines showed modest downregulation in HepG2 cells of the NOX1 gene compared to SH-SY5Y. Despite this, the docking scores of all enzymes were included in the study because off-target effects such as those described above cannot be excluded. However, the obtained docking scores could be indirectly related to pharmacologically or toxicologically relevant biological responses. Indeed, our exploratory analysis showed that strong predicted binding to 5-LOX at the allosteric site was associated with increased neurotoxicity in SH-SY5Y cells, which is likely an off-target effect. While docking correlations must be interpreted cautiously, they provide testable hypotheses suggesting that modifying the 5-LOX interacting motif might reduce cytotoxicity to neuronal cells.

The differential cytoprotective profiles observed between HepG2 and SH-SY5Y cells might be explained by their distinct expression patterns of survival-related genes. We obtained such information from the depmap portal [100,101]. HepG2 cells are liver-derived and have inherently robust antioxidant systems and a high metabolic capacity [102]. According to depmap, they express PGC-1α at significant levels. It can co-activate NRF2 and induce a broad antioxidant response [103,104].

In our experiments, compounds that were potent radical scavengers likely reduced intracellular ROS and possibly had a synergistic effect with the existing strong NRF2-driven responses, thereby preventing oxidative damage in HepG2.

In contrast, SH-SY5Y cells originate from neuronal cells with relatively lower baseline expression of certain antioxidant genes compared to HepG2. Under oxidative stress, they are highly susceptible to membrane damage, followed by apoptosis. SH-SY5Y cells extend neurites and have complex membrane dynamics. Compared to HepG2 cells, they underexpress the extracellular matrix component laminin alpha-5, which might make them more susceptible to membrane stress [105]. Thus, compounds that reinforce membrane stability may provide greater protection under oxidative stress. Our data indicated that cytoprotection in SH-SY5Y correlated better with membrane stabilization effects, observed in the hemolysis assay. Moreover, no positive associations were observed between in vitro neuroprotection and direct radical scavenging.

The anti-apoptotic Bcl-2 protein is a critical regulator of cell death in neuronal cells, and according to depmap, in SH-SY5Y cells, its expression is significantly higher compared to HepG2 cells. It has been shown that neuroprotective agents as resveratrol can rescue SH-SY5Y from oxidative death by upregulating Bcl-2 [106]. Moreover, if a compound stabilizes the cell membrane, it can reduce the trigger for apoptosis [107]. By preventing the loss of mitochondrial membrane potential or the release of cytochrome c, Bcl-2-mediated inhibition of cell death can be aided [108]. This highlights the importance of considering protein expression profiles when interpreting the mechanistic basis of in vitro model systems.

Another interesting observation is that with SH-SY5Y cells, the cytotoxicity rank of compounds could be reversed at different dose levels. For example, it was shown to be inversely related at 50 and 250 µM. This can also be a sign of assay interference as with PAINS alerts, but is reminiscent of hormesis too. Hormesis is a phenomenon in which low doses of a compound can have beneficial or stimulatory effects, but high doses have inhibitory or toxic effects. Many known antioxidants exhibit pro-oxidant or toxic effects at high concentrations. Both vitamin E and vitamin C can promote oxidative damage or cellular toxicity when present in excess due to auto-oxidation or the disturbance of redox balance. The concentration of the antioxidant, its redox potential and the interaction with metal ions are factors which affect its conversion to a pro-oxidant [109]. For example, alpha tocopherol and vitamin C can switch to pro-oxidant effects in the presence of Fe and Cu ions [110]. Thus, further in vivo safety studies might be necessary to obtain information about safe dosing.

Overall, the observed high antioxidant activity of compound **5** is likely due to the presence of a free NH-NH_2_ group, which is much more likely to donate H-atoms than the amino group. The high radical scavenging potential of compounds **5a** and **5d** is related to the presence of a free hydroxyl group in the phenyl residue. The low antioxidant activity of compound **5c** is due to the presence of a methoxy group in the phenyl part of the structure, which likely has a reduced ability to form intermolecular hydrogen bonds. The presence of a free electron pair on the N-atom in the p-dimethylamino fragment of the phenyl residue of compound **5b** is also associated with lower radical scavenging activity.

The major limitation of this applied analysis is the small number of observations in the dataset which hinders the straightforward interpretation of statistical values. This makes the observations to some degree uncertain and only of exploratory nature. For these reasons, the current dataset is not fit for modeling or extracting statistically sound conclusions. Still, a major strength of our study is that it clearly demonstrates how integrating multi-assay antioxidant and cytoprotective screens with in silico analyses can guide compound prioritization. The three methods applied for variable selection provide an exploratory strategy for key variable identification by making use of limited data. Many of our conclusions about solubility, lipophilicity and cytotoxicity resonate with established medicinal chemistry principles, yet we arrived at them through an exploratory data analysis, which demonstrates that robust statistical and machine learning techniques can extract meaningful insights and guide decision-making despite limitations in the dataset and the applied statistical approaches. In a broader context, we exemplify the possibilities for modern cheminformatics-driven lead optimization. Rather than optimizing a compound for a single readout, we simultaneously considered multiple efficacy and liability endpoints, which is more reflective of real-world drug development where polypharmacology and off-target effects determine success.

Our findings can guide variable selection strategies by emphasizing predictors that appear in multiple models. They also suggest tailoring the selection strategy to the experimental context. For assays where models agree poorly as with in vitro neuroprotection, one might need to validate features with additional methods or experiments. In contrast, in contexts with high model agreement, like cytotoxicity studies, one can be more confident in the selected features.

Based on our findings, we propose several specific directions for future optimization and research. First, medicinal chemistry efforts should focus on modulating lipophilicity by introducing polar substituents or reducing overly hydrophobic moieties. For example, the introduction of a hydroxyl donor in oximes can increase polarity, aqueous solubility and metabolic stability while retaining radical scavenging capability [111,112,113]. Increasing TPSA via heteroaromatic substitution is another possibility which could be explored [114]. Still, compounds should remain within an optimal logP range that maximizes cell permeability and efficacy while minimizing non-specific toxicity. This could involve slightly reducing the size of aromatic systems.

More in vitro mechanistic experiments will be necessary to enhance our understanding of how these compounds act at the molecular level. Transcriptomics or proteomics approaches could reveal downstream effectors of cytoprotection or toxicity, guiding further hypothesis-driven modifications. Measuring the effects on antioxidant defense pathways or in vitro enzyme activities in MPO or 5-LOX inhibition assays would clarify the contribution of the proposed mechanisms.

It should be taken into account that all the observations are based on in vitro data and the most promising compound from this series should be evaluated in animal models of oxidative stress-related disease to confirm that its in vitro protective effects could translate to tangible therapeutic benefits.

## 5. Conclusions

This exploratory study profiled a series of novel N-pyrrolyl hydrazide-hydrazones across multiple in vitro assays integrated with in silico property predictions. The main findings highlight that lipophilicity-driven toxicity, membrane permeability and structural alerts are critical considerations for this compound class. Compounds with lower lipophilicity demonstrated a more favorable profile with better radical scavenging and cellular protective effects and lower cytotoxicity. Compound **5a** emerged as a standout neuroprotective agent in the SH-SY5Y cell model, while compounds **5** and **5d** were balanced, combining strong antioxidant capacity with hepatoprotection in HepG2 cells. It was shown that the observed in vitro hepatoprotective effects were associated with radical scavenging, and in vitro neuroprotective effects were associated with membrane stabilization. Rational design guided by the factors illuminated in this study—lipophilicity, hydrogen bonding and the avoidance of promiscuous motifs—should enhance the safety and effectiveness of new candidates. Ultimately, our data-driven approach sets a foundation for refining these in vitro findings into a viable in vivo therapeutic strategy against oxidative stress-related damage.

## Figures and Tables

**Table 1:** A summary of variable groups by experiment type. Column “MFA Active” indicates whether the group of variables should actively participate in axes formation or should only be used as supplementary, providing context for the interpretation of active variables. Each group of variables contains either only quantitative or qualitative information. The numbers appended at the end of each variable’s name indicate treatment concentrations.

Group	MFA Active	Type	Var. Count	Contained Variables
antiox	Yes	Quanti.	6	ABTS_antioxidant_31, ABTS_antioxidant_125, ABTS_antioxidant_250, DPPH_antioxidant_31, DPPH_antioxidant_125, DPPH_antioxidant_250
protect_SH-SY5Y	Yes	Quanti.	6	SH-SY5Y_protect_0.01, SH-SY5Y_protect_0.1, SH-SY5Y_protect_0.5, SH-SY5Y_protect_1, SH-SY5Y_protect_5, SH-SY5Y_protect_10
protect_HepG2	Yes	Quanti.	4	HepG2_protect_0.1, HepG2_protect_1, HepG2_protect_10, HepG2_protect_20
cytotox_HepG2	Yes	Quanti.	8	HepG2_cytotox_1, HepG2_cytotox_5, HepG2_cytotox_10, HepG2_cytotox_50, HepG2_cytotox_75, HepG2_cytotox_100, HepG2_cytotox_250, HepG2_cytotox_500
cytotox_SH-SY5Y	Yes	Quanti.	8	SH-SY5Y_cytotox_1, SH-SY5Y_cytotox_5, SH-SY5Y_cytotox_10, SH-SY5Y_cytotox_25, SH-SY5Y_cytotox_50, SH-SY5Y_cytotox_75, SH-SY5Y_cytotox_100, SH-SY5Y_cytotox_250
hemolysis_erythrocytes	Yes	Quanti.	3	erythrocytes_hemolysis_25, erythrocytes_hemolysis_50, erythrocytes_hemolysis_100
dock	No	Quanti.	5	Cyp_2E1_docking, LOX_act_docking, LOX_allost_docking, MPO_docking, NOX_docking
ADME.quali	No	Quali.	11	esol_class, ali_class, silicos.it_class, gi_absorption, bbb_permeant, pgp_substrate, cyp1a2_inhibitor, cyp2c19_inhibitor, cyp2c9_inhibitor, cyp2d6_inhibitor, cyp3a4_inhibitor
ADME.quanti	No	Quanti.	16	esol_log_s, esol_solubility_.mg.ml., esol_solubility_.mol.l., ali_log_s, ali_solubility_.mg.ml., ali_solubility_.mol.l., silicos.it_logsw, silicos.it_solubility_.mg.ml., silicos.it_solubility_.mol.l., ilogp, xlogp3, wlogp, mlogp, silicos.it_log_p, consensus_log_p, log_kp_.cm.s.
chem.quanti	No	Quanti.	9	mw, X.heavy_atoms, X.aromatic_heavy_atoms, fraction_csp3, X.rotatable_bonds, X.h.bond_acceptors, X.h.bond_donors, mr, TPSA
dr_des_quanti	No	Quanti.	10	lipinski_.violations, ghose_.violations, veber_.violations, egan_.violations, muegge_.violations, bioavailability_score, pains_.alerts, brenk_.alerts, leadlikeness_.violations, synthetic_accessibility

**Table 2:** Summary of clusters after consolidation step in HCPC from MFA-analyzed data and leading associations with quantitative variables.

Cluster	Variable	v-Test	Mean in Cluster	Overall Mean	*p*-Value
Cluster 1: **c5a**	SH.SY5Y_protect_5	2.41	70.6	8.36	0.02
	SH.SY5Y_protect_10	2.33	80.07	9.56	0.02
	SH.SY5Y_protect_1	2.24	42.4	2.76	0.02
Cluster 2: **c5g**	LOX_allost_docking	2.03	8.03	6.96	0.04
	MPO_docking	1.99	9.49	8.07	0.05
	HepG2_cytotox_500	1.99	98.84	96.63	0.05
Cluster 3: **c5b**, **c5c**, **c5f**	X.h.bond_donors	−2.45	1	1.57	0.01
Cluster 4: **c5**, **c5d**	DPPH_antioxidant_250	2.42	32.93	8.2	0.02
	DPPH_antioxidant_125	2.41	19.93	0.78	0.02
	DPPH_antioxidant_31	2.32	5.34	−5.14	0.02
	ABTS_antioxidant_250	2.31	81.09	22.53	0.02
	ABTS_antioxidant_31	2.27	21.72	1.5	0.02
	ABTS_antioxidant_125	2.22	70.81	18.38	0.03
	HepG2_protect_1	2.12	47.49	37.09	0.03
	HepG2_protect_0.1	2.06	42.31	28.56	0.04
	log_kp_.cm.s.	−2.03	−5.8	−5.39	0.04

## Data Availability

R code for data analysis is available on reasonable request from the authors. For data requests, email yyordanov@pharmfac.mu-sofia.bg.

## Supplementary Materials

The following supporting information can be downloaded at https://www.mdpi.com/article/10.3390/antiox14050566/s1, Supplementary Materials S1—in_vitro_in_silico_data_generation.docx (Supplementary References [115,116,117,118,119,120,121,122,123,124,125,126,127,128,129,130,131,132,133]); Supplementary Materials S2—in_vitro_data_summary.csv; Supplementary Materials S3—full_dataset_wide_format.csv. Figure S1. Structure of in vitro and docking data, used for the multiple factor analysis. Plot, generated with R packages data.tree [115] and DiagrammeR [116]. Figure S2. Human gastrointestinal absorption and blood-brain barrier permeation and PGP-substrate prediction, represented by a BOILED-Egg diagram. Blue circles represent predicted PGP substrates and red circles—non-substrates. Figure S3. Binding sites of the studied enzymes A. MP, B. NOX, C. CYP2E1, D. LOX active binding site, E. LOX allosteric binding site, F. COX-2, G. COX-1; amino acids (AA), interacting with the ligand and overlay of the corresponding native ligand structures (yellow) and redocking con-formations (grey). Figure S4. Binding affinities to MP, NOX, CYP2E1, LOX active binding site, LOX allosteric binding site, COX-2 and COX-1, calculated by AutoDock Vina of test compounds, co-crystallized ligand controls, known inhibitor controls and known natural compounds with pleiotropic antioxidant effects. Only the conformations with best binding affinity and similar (RMSD u.b. < 6) were plotted. Figure S5. Interaction of the test compounds, co-crystallized ligand controls, known inhibitor controls and known natural compounds with pleiotropic antioxidant effects with MPO. Arrows represent shared amino acid residue interactions with controls. Figure S6. Interaction of the test compounds, co-crystallized ligand controls, known inhibitor controls and known natural compounds with pleiotropic antioxidant effects with NOX. Arrows represent shared amino acid residue interactions with controls. Figure S7. Interaction of the test compounds, co-crystallized ligand controls, known inhibitor controls and known natural compounds with pleiotropic antioxidant effects with 5-LOX active binding site. Arrows represent shared amino acid residue interactions with controls. Figure S8. Interaction of the test compounds, co-crystallized ligand controls, known inhibitor controls and known natural compounds with pleiotropic antioxidant effects with 5-LOX allosteric binding site. Arrows represent shared amino acid residue interactions with controls. Figure S9. Interaction of the test compounds, co-crystallized ligand controls, known inhibitor controls and known natural compounds with pleiotropic antioxidant effects with COX-2. Arrows represent shared amino acid residue interactions with controls. Table S1. Description of SwissADME-generated in silico parameters, related to solubility and ADME. Table S2. Macromolecule structure properties and binding site grid coordinates. 2-(3,5-bistrifluoromethylbenzylamino)-6-oxo-1H-pyrimidine-5-carbohydroxamic acid (NIH). Table S3. Automated report about the used R programming environment and all relevant package versions. Table S4. Selected predictors by three methods, based on lasso (L), Huber robust regression (R), and random forest (RF). Predictor variables selected by a consensus of three methods were pre-sented in blue and those selected by two methods—in orange.

## Abbreviations

The following abbreviations are used in this manuscript:

antiox	Radical scavenging antioxidant assay, such as ABTS or DPPH;
cytotox	Cytotoxicity assay, such as 3-(4,5-dimethylthiazol-2-yl)-2,5-diphenyltetrazolium bromide assay;
hemol	Hemolysis assay with human erythrocytes;
protect	Assay for protective effects on hydrogen peroxide-damaged cell culture;
dock	Vina docking affinity;
ADME	Absorption, distribution, metabolism and excretion—related SwissADME predictor variables: gi_absorption, bbb_permeant, pgp_substrate, cyp1a2_inhibitor, cyp2c19_inhibitor, cyp2c9_inhibitor, cyp2d6_inhibitor, cyp3a4_inhibitor, log_kp_.cm.s.;
chem	Molecular size-related SwissADME predictor variables: mw, X.heavy_atoms, X.aromatic_heavy_atoms, fraction_csp3, X.rotatable_bonds, X.h.bond_acceptors, X.h.bond_donors, mr, TPSA;
lip_sol	Lipophyllicity-related SwissADME predictor variables: ilogp, xlogp3, wlogp, mlogp, silicos.it_log_p, consensus_log_p;
wat_sol	Water solubility-related SwissADME predictor variables: esol_log_s, esol_solubility_.mg.ml., esol_solubility_.mol.l., esol_class, ali_log_s, ali_solubility_.mg.ml., ali_solubility_.mol.l., ali_class, silicos.it_logsw, silicos.it_solubility_.mg.ml., silicos.it_solubility_.mol.l., silicos.it_class;
lead_likeness	Lead-likeness-related SwissADME predictor variables: leadlikeness_.violations, synthetic_accessibility;
drug_likeness	Drug-likeness-related SwissADME predictor variables: lipinski_.violations, ghose_.violations, veber_.violations, egan_.violations, muegge_.violations, bioavailability_score;
med_chem_alerts	SwissADME predictor variables for filtering potentially problematic molecular fragments: pains_.alerts, brenk_.alerts;
drug_design	A summary term for the following type of SwissADME variables for filtering out molecules with problematic properties for drug design: lead_likeness, drug_likeness and med_chem_alerts;
mw	Molecular weight;
mr	Molar refractivity;
TPSA	Topological polar surface area;
fraction_csp3	Proportion of sp3-hybridized carbons in a molecule;
LogP	Logarithm of the partition coefficient between 1-octanol and water.

Further descriptions of predictor parameters can be found in Supplementary Table S1.
