# Supplementary material for: Exploratory Data Analysis of the In Vitro Effects of Novel Hydrazide-Hydrazone Antioxidants in the Context of In Silico Predictors
Source: Antioxidants (Basel). 2025 May 8;14(5):566. doi: 10.3390/antiox14050566 (PMC12108285; doi:10.3390/antiox14050566)
Supplement: Supplementary file 1 [file antioxidants-14-00566-s001.zip › Supplementary Materials S1-in_vitro_in_silico_data_generation.pdf]

antioxidants

MDPI

Supplementary Materials S1

Supplementary Methods

In vitro safety and efficacy data

The DPPH and ABTS assays have been applied to the series of compounds as previously described [1]. Briefly, the DPPH assay has been applied to assess the radical scavenging ability of the compounds by measuring their capacity to neutralize 2,2-diphenyl-1-picrylhydrazyl radicals. Similarly, the ABTS assay the reducing power of the hydrazones has been determined by measuring their interaction with ABTS•+ radicals. Cytotoxicity and protective effects have been assessed through cell-based assays on HepG2 and SH-SY5Y cells using the MTT-dye reduction method [2]. To evaluate hemocompatibility, a hemolysis assay has been conducted on human erythrocytes [1]. The in vitro results data, included in the analyzed dataset is summarized in Figure S1.

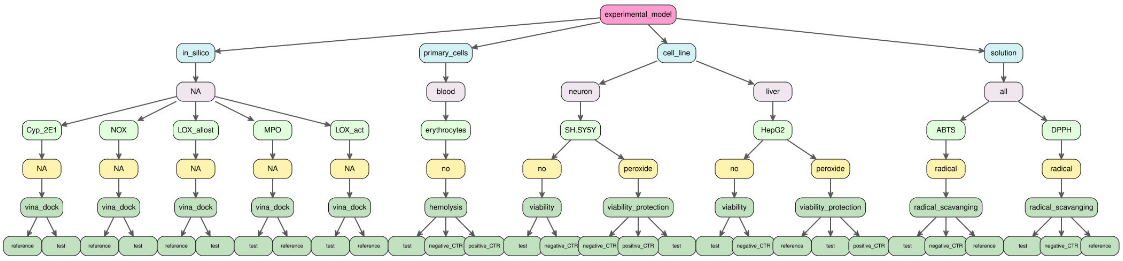

Figure S1. Structure of in vitro and docking data, used for the multiple factor analysis. Plot, generated with R packages data.tree [3] and DiagrammeR [4].

ADME-related predictions

We applied the SwissADME web tool (http://www.swissadme.ch/, accessed 05.10.2024) [5,6] on the series of compounds. The tool predicts a series of parameters (Table S1), based on smiles input with major significance of the log Po/w and polar surface area (PSA) for predicting passive diffusion through both physiological barriers human gastrointestinal absorption (HIA)(trained on 660 compounds) and blood-brain barrier (BBB)(trained on 260 compounds)[7]. This tool also predicts whether the compound is a substrate of multidrug resistance protein 1 also known as P-glycoprotein 1 (PGP) [6]. The propensity of test molecules to be inhibitors of the major drug-metabolizing enzyme isoforms (CYP1A2, CYP2C19, CYP2C9, CYP2D6, CYP3A4) was calculated by SwissADME. Its predictions are based on a support vector machine algorithm was also trained on datasets of established substrates/non-substrates and inhibitors/non-inhibitors [1]. The training sets include data on interactions with CYP1A2 (4301 inhibitors, 4844 non-inhibitors), CYP2C19 (4284 inhibitors, 4988 non-inhibitors); CYP2C9 (2940 inhibitors, 3000 non-inhibitors); CYP2D6 (1814 inhibitors, 1850 non-inhibitors); CYP3A4 (3758 inhibitors, 3760 non-inhibitors) [5].

Table S1. Description of SwissADME-generated in silico parameters, related to solubility and ADME.

Parameter	Description
**Physicochemical Properties**	
canonical_smiles	The SMILES (Simplified Molecular Input Line Entry System) representation of the compound's chemical structure.
formula	The molecular formula of the compound.
mw	Molecular weight.
X.heavy_atoms	Number of heavy (non-hydrogen) atoms.
X.aromatic_heavy_atoms	Number of aromatic heavy atoms.
fraction_csp3	Fraction of sp3 hybridized carbons.
X.rotatable_bonds	Number of rotatable bonds.
X.h.bond_acceptors	Number of hydrogen bond acceptors.
X.h.bond_donors	Number of hydrogen bond donors.
mr	Molar refractivity.
tpsa	Topological polar surface area.
ilogp, xlogp3, wlogp, mlogp, silicos.it_log_p, consensus_log_p	Various predicted logP values, which are used to measure the hydrophobicity/lipophilicity of the compounds.
canonical_smiles	The SMILES (Simplified Molecular Input Line Entry System) representation of the compound's chemical structure.
formula	The molecular formula of the compound.
mw	Molecular weight.
X.heavy_atoms	Number of heavy (non-hydrogen) atoms.
X.aromatic_heavy_atoms	Number of aromatic heavy atoms.
fraction_csp3	Fraction of sp3 hybridized carbons.
X.rotatable_bonds	Number of rotatable bonds.
X.h.bond_acceptors	Number of hydrogen bond acceptors.
**Solubility Predictions**	
esol_log_s, esol_solubility_.mg.ml., esol_solubility_.mol.l., esol_class	ESOL (Estimated Solubility) predictions and solubility classifications.
ali_log_s, ali_solubility_.mg.ml., ali_solubility_.mol.l., ali_class	Solubility predictions from the Ali et al. method.
silicos.it_logsw, silicos.it_solubility_.mg.ml., silicos.it_solubility_.mol.l., silicos.it_class	Solubility predictions from the Silicos-IT model.
**ADMET Predictions**	
gi_absorption	Gastrointestinal absorption prediction.
bbb_permeant	Prediction of blood-brain barrier permeability.
pgp_substrate	Whether the compound is a substrate for P-glycoprotein.
cyp1a2_inhibitor, cyp2c19_inhibitor, cyp2c9_inhibitor, cyp2d6_inhibitor, cyp3a4_inhibitor	Predictions of inhibition for various cytochrome P450 enzymes (important in drug metabolism).
log_kp_.cm.s.	Skin permeability prediction.
gi_absorption	Gastrointestinal absorption prediction.
bbb_permeant	Prediction of blood-brain barrier permeability.
pgp_substrate	Whether the compound is a substrate for P-glycoprotein.
cyp1a2_inhibitor, cyp2c19_inhibitor, cyp2c9_inhibitor, cyp2d6_inhibitor, cyp3a4_inhibitor	Predictions of inhibition for various cytochrome P450 enzymes (important in drug metabolism).
log_kp_.cm.s.	Skin permeability prediction.
gi_absorption	Gastrointestinal absorption prediction.
bbb_permeant	Prediction of blood-brain barrier permeability.
**Drug-Likeness**	
lipinski_.violations, ghose_.violations, veber_.violations, egan_.violations, muegge_.violations	These columns indicate violations of various drug-likeness rules (e.g., Lipinski's Rule of Five).
bioavailability_score	A predicted bioavailability score.
pains_.alerts	Alerts for Pan-Assay Interference Compounds (PAINS), which can be problematic in drug discovery.
brenk_.alerts	Brenk's structural alerts for drug-likeness.
leadlikeness_.violations	Violations of lead-likeness criteria.
synthetic_accessibility	A score indicating how easily the compound can be synthesized.
lipinski_.violations, ghose_.violations, veber_.violations, egan_.violations, muegge_.violations	These columns indicate violations of various drug-likeness rules (e.g., Lipinski's Rule of Five).
bioavailability_score	A predicted bioavailability score.
pains_.alerts	Alerts for Pan-Assay Interference Compounds (PAINS), which can be problematic in drug discovery.
brenk_.alerts	Brenk's structural alerts for drug-likeness.
leadlikeness_.violations	Violations of lead-likeness criteria.
synthetic_accessibility	A score indicating how easily the compound can be synthesized.
lipinski_.violations, ghose_.violations, veber_.violations,	These columns indicate violations of various drug-likeness rules (e.g., Lipinski's Rule of Five).

egan_.violations, muegge_.violations	

Ligand and protein structure preprocessing

Ligand structures and 3D conformations were generated in Marvin Sketch (version 21.6) for the test molecules or downloaded from ZINC database [8] for reference molecules and controls. The generated structures in SDF format were then converted to PDBQT file format by OpenBabel software (version 2.4.1) with only polar hydrogens and charges added [9].

Five prooxidant enzymes were selected and their structures obtained from the Protein Data Bank (PDB, https://www.rcsb.org) [10]. The criteria for PDB entries selection included a preference for crystallographic structures, obtained by X-ray diffraction, resolution better than 3Å, inhibitor-bound, small molecule ligands (< 900 daltons [11]), preference for human or mammal origin. The macromolecule structures were then preprocessed in Autodock Tools (version 1.5.6) by removing water molecules and co-crystallized ligand atoms at the selected binding site, adding only polar hydrogens, introducing charges and converting to PDBQT file format.

Molecular docking

The structure of docking results is summarized in Figure S2. Docking coordinates were selected either by a literature search or by generation of a box, centered on the co-crystallized ligand, fully covering the surrounding binding pocket (Table S2). Binding site coordinate box selection was done in AutoDock Vina (version 1.2.0), followed by a validation step of redocking [12,13].

Table S2. Macromolecule structure properties and binding site grid coordinates. 2-(3,5-bistrifluoromethylbenzylamino)-6-oxo-1H-pyrimidine-5-carbohydroxamic acid (NIH).

PDB entry	Crystallographic method	Resolution	Grid centre	Grid size	Cocrystallized ligand
NOX (2CDU) [14]	x-ray diffraction	1.80 Å	1.687x 9.885y 54.962z	30x 14y 32z	adenosine-5'-diphosphate
MPO (5c1M) [15]	x-ray diffraction	2.00 Å	24.123x 3.191y 43.257z	24x 24y 24z	NIH
CYP2E1 (3E5f) [16]	x-ray diffraction	2.60 Å	5.315x -3.953y 35.984z	20x 20y 20z	4-methylpyrazole
5-LOX-act. site (6N2W) [17]	x-ray diffraction	2.71 Å	36.057x 65.306y 39.198z	20x 22y 30z	nordihydroguaiaretic acid
5-LOX allos. site (6NCF) [18]	x-ray diffraction	2.87 Å	11.516x 22.609y -18.912z	24x 22y 22z	3-acetyl-11-keto-beta-boswellic acid
COX2 (3LN1)	x-ray diffraction	2.40 Å	30x -22y	22x 22y	Celecoxib

[19]			-16z	22z	
COX1<br>(3N8Z)<br>[20]	x-ray diffraction	2.90 Å	19.8x<br>48y<br>10z	22x<br>22y<br>22z	Flurbiprofen

Macromolecule-ligand interactions were calculated by a docking simulation in AutoDock Vina (version 1.2.0) [12,13] and only conformations with favorable affinity (negative free binding energy) values, comparable to the co-crystallized compound or known inhibitor drug were further prioritized and evaluated for intermolecular interactions.

Graphic representation

Docking results, namely binding energies of the best conformation for each ligand and favorable conformations (up to an RMSD value from the best conformation) were created by the open-source data visualization package ggplot2 (version 3.5.1) [21] running on the integrated development environment R Studio (version 2024.12.1.563) [22] for the statistical programming language R (version 4.4.3) [23]. Intermolecular interactions were plotted in BIOVIA Discovery Studio Visualizer (version 21.1.0.20298) [24].

R programming environment and package versions

Table S3. Automated report about the used R programming environment and all relevant package versions.

R version	4.4.3 (2025-02-28)
Platform	x86_64-pc-linux-gnu
Running under	Ubuntu 24.04.2 LTS
Matrix products	default
BLAS	/usr/lib/x86_64-linux-gnu/blas/libblas.so.3.12.0
LAPACK	/usr/lib/x86_64-linux-gnu/lapack/liblapack.so.3.12.0
locale	LC_CTYPE=en_US.UTF-8
	LC_NUMERIC=C
	LC_TIME=en_US.UTF-8
	LC_COLLATE=en_US.UTF-8
	LC_MONETARY=en_US.UTF-8
	LC_MESSAGES=en_US.UTF-8
	LC_PAPER=en_US.UTF-8
	LC_NAME=en_US.UTF-8
	LC_ADDRESS=en_US.UTF-8
	LC_TELEPHONE=en_US.UTF-8
	LC_MEASUREMENT=en_US.UTF-8
	LC_IDENTIFICATION=en_US.UTF-8
time zone	Europe/Sofia
tzcode source	system (glibc)
attached base packages	grid, stats, graphics, grDevices, utils, datasets, methods, base
other attached packages	ggpubr_0.6.0, magick_2.8.6, rsvg_2.6.2, DiagrammeRsvg_0.1, DiagrammeR_1.0.11, data.tree_1.1.0, mixOmics_6.30.0, plotly_4.10.4, rcdk_3.8.1, rcdklibs_2.9, rJava_1.0-11, random-Forest_4.7-1.2, cluster_2.1.8.1, BiocManager_1.30.25, corrplot_0.95, factoextra_1.0.7, FactoMineR_2.11, pheatmap_1.0.12, writexl_1.5.2,

	here_1.0.1, patchwork_1.3.0, gridExtra_2.3, robustbase_0.99-4-1, car_3.1-3, carData_3.0-5, boot.pval_0.7.0, ggtext_0.1.2, kableEx-tra_1.4.0, ggforce_0.4.2, reshape2_1.4.4, UpSetR_1.4.0, MASS_7.3-65, ti-dytext_0.4.2, ggrepel_0.9.6, pls_2.8-5, caret_7.0-1, lat-tice_0.22-6, steprf_1.0.2, hqreg_1.4-1, glmnet_4.1-8, Matrix_1.7-3, Hmisc_5.2-3, ggcorrplot_0.1.4.1, lubridate_1.9.4, forcats_1.0.0, stringr_1.5.1, dplyr_1.1.4, purrr_1.0.4, readr_2.1.5, tidyr_1.3.1, tib-ble_3.2.1, ggplot2_3.5.1, tidyverse_2.0.0
loaded via a namespace (and not attached)	splines_4.4.3, polyclip_1.10-7, hardhat_1.4.1, pROC_1.18.5, rpart_4.1.24, lifecycle_1.0.4, rstatix_0.7.2, Rdpack_2.6.3, rpro-jroot_2.0.4, globals_0.16.3, flashClust_1.01-2, back-ports_1.5.0, SnowballC_0.7.1, magrittr_2.0.3, sass_0.4.9, rmarkdown_2.29, jquerylib_0.1.4, yaml_2.3.10, fin-gerprint_3.5.7, RColor-Brewer_1.1-3, multcomp_1.4-28, abind_1.4-8, pkgload_1.4.0, iter-tools_0.1-3, nnet_7.3-20, TH.data_1.1-3, tweenr_2.0.3, sand-wich_3.1-1, ipred_0.9-15, lava_1.8.1, tokenizers_0.3.0, lis-tenv_0.9.1, el-lipse_0.5.0, RSpectra_0.16-2, parallelly_1.42.0, svglite_2.1.3, commonmark_1.9.5, codetools_0.2-20, DT_0.33, xml2_1.3.8, tidyselect_1.2.1, shape_1.4.6.1, farver_2.1.2, matrix-Stats_1.5.0, stats4_4.4.3, base64enc_0.1-3, json-lite_1.9.1, For-mula_1.2-5, survival_3.8-3, iterators_1.0.14, emmeans_1.11.0, sys-temfonts_1.2.1, foreach_1.5.2, tools_4.4.3, ragg_1.3.3, rARPACK_0.11-0, Rcpp_1.0.14, glue_1.8.0, prodlim_2024.06.25, xfun_0.51, withr_3.0.2, fastmap_1.2.0, boot_1.3-31, digest_0.6.37, timechange_0.3.0, R6_2.6.1, estimability_1.5.1, textshaping_1.0.0, colorspace_2.1-1, markdown_1.13, generics_0.1.3, corpcor_1.6.10, data.table_1.17.0, recipes_1.2.0, class_7.3-23, httr_1.4.7, html-widgets_1.6.4, scatterplot3d_0.3-44, ModelMetrics_1.2.2.2, pkgconfig_2.0.3, gtable_0.3.6, timeDate_4041.110, janeaus-tenr_1.0.0, htmltools_1.5.8.1, multcompView_0.1-10, scales_1.3.0, png_0.1-8, leaps_3.2, spm_1.2.2, gower_1.0.2, knitr_1.50, rstudi-oapi_0.17.1, tzdb_0.5.0, curl_6.2.1, visNetwork_2.1.2, check-mate_2.3.2, nlme_3.1-167, cachem_1.1.0, zoo_1.8-13, parallel_4.4.3, foreign_0.8-88, pillar_1.10.1, vctrs_0.6.5, xtable_1.8-4, htmlTa-ble_2.4.3, evaluate_1.0.3, mvtnorm_1.3-3, cli_3.6.4, compiler_4.4.3, rlang_1.1.5, crayon_1.5.3, ggsignif_0.6.4, future.apply_1.11.3, la-beling_0.4.3, plyr_1.8.9, stringi_1.8.4, BiocPar-allel_1.40.0, viridis-Lite_0.4.2, munsell_0.5.1, lazyeval_0.2.2, V8_6.0.2, hms_1.1.3, fu-ture_1.34.0, rbibu-tils_2.3, gridtext_0.1.5, broom_1.0.7, igraph_2.1.4, bslib_0.9.0, DEoptimR_1.1-3-1

Supplementary In Silico Results

In vitro safety and efficacy data

A summary of the in vitro data, used for the creation of the dataset is available in the "Supplementary_S2_in_vitro_summary.csv" file.

In silico membrane permeation prediction

Using the SwissADME engine, according to compounds' WLOGP and TPSA values, a BoiledEGG plot was generated, which represents the predicted propensity of molecules

to cross the intestinal and blood-brain barriers and probable PGP substrates were indicated (Figure S1). According to this model, none of the test compounds 5-5g permeate the blood-brain barrier and most of them are located near the threshold between absorbed and non-absorbed substances by the gastrointestinal tract (GIT) except for 3, which was predicted to be well into the domain of orally bioavailable substances. Compound 5d is the only substance clearly outside of this domain. Substances 5a-g have similar properties to celecoxib in this regard. However, celecoxib was predicted not to be a PGP substrate unlike compounds 5a-5f. Compounds 5 and 5g were predicted not to be PGP substrates. Among the tested reference structures, only flurbiprofen, SNB, clomethiazole and CAPE were predicted not to be PGP substrates and to cross the blood-brain barrier. It was predicted that quercetin, curcumin and zileuton are not PGP substrates and are absorbed through GIT, but do not to cross the BBB.

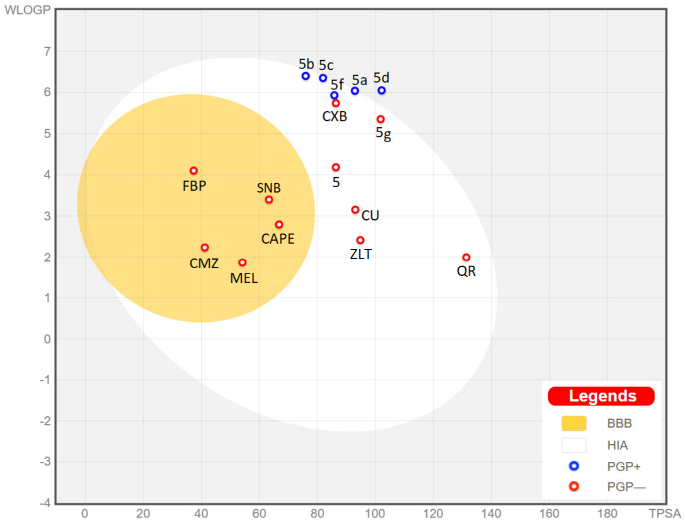

Figure S2. Human gastrointestinal absorption and blood-brain barrier permeation and PGP-substrate prediction, represented by a BOILED-Egg diagram. Blue circles represent predicted PGP substrates and red circles – non-substrates.

CYP450 inhibition prediction

SwissADME predicted that all compounds 5-5g are probably inhibitors of CYP450 isoforms 2C19, 2C9 and 3A4. Compound 5b was predicted to also inhibit CYP2D6 and compound 5g to inhibit CYP1A2.

Evaluation of molecular docking

There was good overlap with low RMSD values between the X-ray crystallographic data ligand poses (yellow, Figure S4.) and the AutoDock Vina-calculated ligand pose (grey, purple and red, Figure S3.).

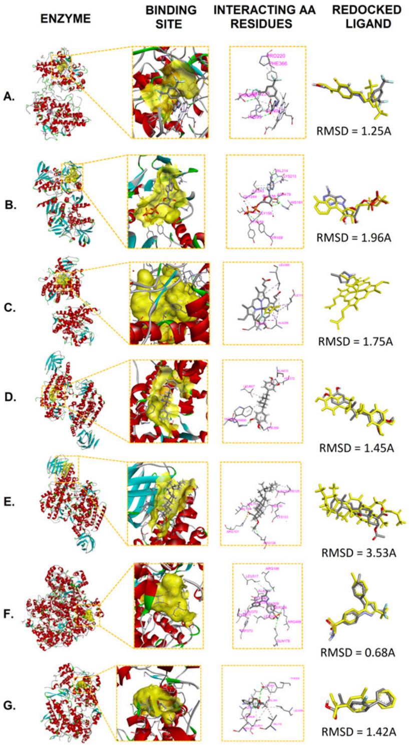

Figure S3. Binding sites of the studied enzymes A. MP, B. NOX, C. CYP2E1, D. LOX active binding site, E. LOX allosteric binding site, F. COX-2, G. COX-1; amino acids (AA), interacting with the ligand and overlay of the corresponding native ligand structures (yellow) and redocking conformations (grey).

An indication that the chosen protein structures are adequate for docking studies was that all control structures have negative binding energies, the least favorable binding energy being -4 kcal/mol between 4PZ and CYP2E1 and the most favorable binding energy being -11.7 kcal/mol between celecoxib and COX-2. In all other instances the binding energies of inhibitor controls were of a relatively high magnitude, between -7 to -9 kcal/mol, suggesting that there is affinity between protein and ligand (Figure S4.).

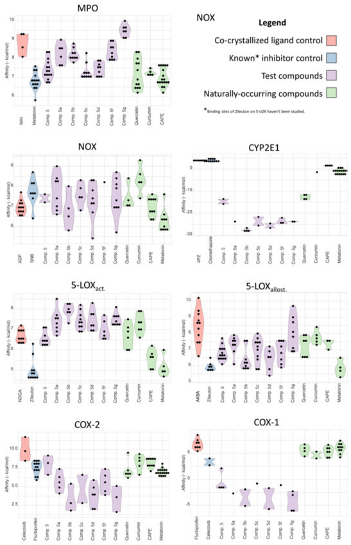

Figure S4. Binding affinities to MP, NOX, CYP2E1, LOX active binding site, LOX allosteric binding site, COX-2 and COX-1, calculated by AutoDock Vina of test compounds, co-crystallized ligand controls, known inhibitor controls and known natural compounds with pleiotropic antioxidant effects. Only the conformations with best binding affinity and similar (RMSD u.b.<6) were plotted.

MPO

Compounds 5-5g had similar predicted affinities to MPO compared to the both endogenous and known inhibitor controls, as well as to the tested natural compounds. Compound 5g had the strongest affinity, superior to that of all test compounds, natural compounds and inhibitor controls. Compounds 5a, 5b and 5f had superior affinities than that of melatonin, quercetin, curcumin and CAPE and comparable to the redocked co-crystallized inhibitor 2-(3,5-bistrifluoromethylbenzylamino)-6-oxo-1H-pyrimidine-5-carbohydroxamic acid (NIH) (Figure S5.). There are three MPO binding site amino acid interactions in common between NIH and melatonin: PHE D:336, ARG D:239 and HEM B:605. The compounds with strongest binding affinities 5a, 5b, 5f and 5g were predicted to interact with PHE D:336, ARG D:239 and HEM B:605 like both NIH and melatonin, and with GLU B:102 like only melatonin was predicted to do. Compounds 5a and 5b were also predicted to interact with HIS B:95 like only NIH does.

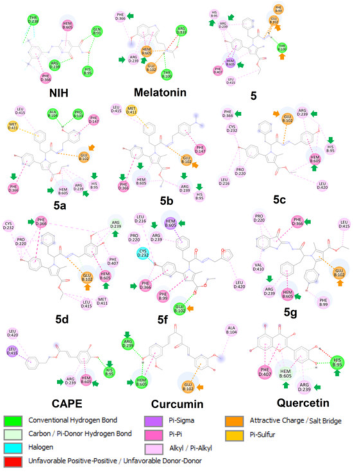

Figure S5. Interaction of the test compounds, co-crystallized ligand controls, known inhibitor controls and known natural compounds with pleiotropic antioxidant effects with MPO. Arrows represent shared amino acid residue interactions with controls.

NOX

In the case of NOX, the known inhibitor control SNB was predicted to have a more favorable affinity to the binding site than the redocked co-crystallized ligand ADP. Among the test compounds, all except compound 5 showed superior predicted binding affinities than CAPE and melatonin, comparable to quercetin, but lower than that of curcumin. Curcumin stands out with the best predicted affinity among all docked structures. Compound 5a, followed by compound 5g were predicted to have slightly better affinities, compared to SNB and slightly lower than curcumin's (Figure S6.).

There are four common interactions with amino acids at the NOX binding site between SNB and ADP, namely TYR159, ILE160, ASP179 and TYR188. Compounds 5a, 5d and 5f were predicted to interact only with TYR188 like ADP, but unlike SNB and for compounds 5c and 5g - with TYR159 and ILE160 like both ADP and SNB, with TYR181 and HIS181 like only ADP does and with FADD, like only SNB does (Figure S6.).

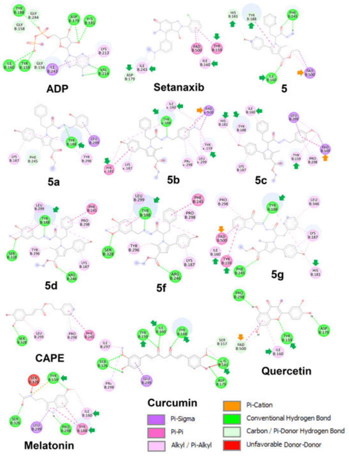

Figure S6. Interaction of the test compounds, co-crystallized ligand controls, known inhibitor controls and known natural compounds with pleiotropic antioxidant effects with NOX. Arrows represent shared amino acid residue interactions with controls.

5-LOX

Docking simulations for predicting 5-LOX affinity were done at two protein binding sites – the iron-containing active binding site, inhibited by NDGA and the allosteric site – inhibited by AKBA (Figures S1.8. and S1.9.). All test compounds had better predicted affinities to the active binding site of 5-LOX, compared to zileuton and except for compound 3, a better affinity than co-crystallized nordihydroguaiaretic acid (NDGA). Curcumin and quercetin also have better affinities than NDGA, but slightly lower affinities than compounds 5a-g. CAPE and melatonin have comparable affinities to zileuton, but lower affinities than NGDA. All tested compounds were predicted to have some affinity to the allosteric binding site of 5-LOX, which is comparable to Zileuton's, but lower than the endogenous ligand control – 3-acetyl-11-keto-beta-boswellic acid (AKBA), except for compound 5g, which stands out for its high affinity, comparable to AKBA's (Figure S5.).

Three ligand-amino acid interactions are common between 5-LOX active site inhibitor NDGA and zileuton: LEU607, HIS372 and TRP599 (Figure S8.). Compound 5a was predicted to interact with LEU607 and HIS372 like both controls, with PHE359 like only NDGA does with ALA603, HIS367 and LEU368 like only zileuton does. Compound 5b was predicted to interact with HIS372 like both NDGA and zileuton, with PHE359 and ALA410 like NDGA and with ALA603, HIS367 and LEU368 like zileuton. Compound 5c was indicated to interact with the same amino acid residues as compound 5b, but without HIS367 and 5d to interact with the same amino acid residues as compound 5c, but instead of HIS372, it interacts with TRP599, which is also common for NDGA and zileuton. Compound 5g interacts with HIS372 like both NDGA and zileuton, with ALA410, like only NGDA does and with LEU368 – like only zileuton does. Curcumin interacts with HIS372 and LEU607 like both NDGA and zileuton, with PHE359, ALA410 and HIS600, like NDGA and with GLN363, ALA603 and LEU368, like zileuton. Among those interactions with the active binding site, similar between test molecules and known inhibitors are one hydrogen bond with compounds 5a and 5f and two hydrogen bonds with compound 5g (Figure S7.).

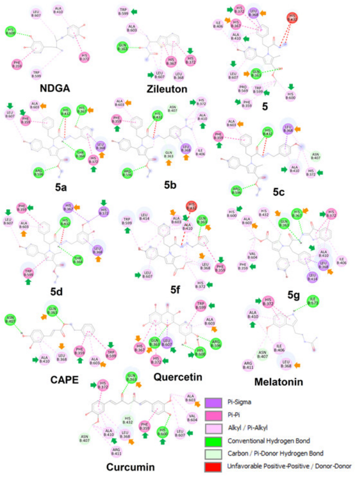

Figure S7. Interaction of the test compounds, co-crystallized ligand controls, known inhibitor controls and known natural compounds with pleiotropic antioxidant effects with 5-LOX active binding site. Arrows represent shared amino acid residue interactions with controls.

There are four amino acid residues at the 5-LOX allosteric binding site that both AKBA and zileuton interact with: HIS130, ARG101, VAL110 and LYS133 (Figure S8.). Compounds 5a, 5b, 5f and 5g all interact with these same four amino acid residues, but like only AKBA does, compounds 5a, 5b and 5g interact also with ARG138. Compound 5g also interacts with LEU66 like zileuton and unlike AKBA. Regarding interaction with 5-LOX allosteric site, the similarities between test molecules and known inhibitors are: one hydrogen bond in common with compound 5a, two hydrogen bonds with compounds 5b and 5f and three hydrogen bonds with compound 5g.

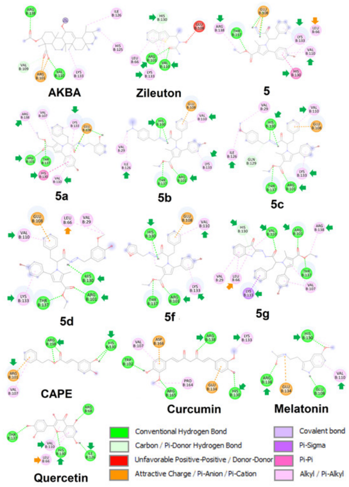

Figure S8. Interaction of the test compounds, co-crystallized ligand controls, known inhibitor controls and known natural compounds with pleiotropic antioxidant effects with 5-LOX allosteric binding site. Arrows represent shared amino acid residue interactions with controls.

COX-2

Docking studies of COX-2 showed that all of the test compounds and natural compound structures may have affinities to the specified binding site much lower than that of co-crystallized control compound celecoxib (Figure S4.). Only compound 5 was predicted to have an affinity close to that of the tested natural compounds and the selective COX-1 inhibitor flurbiprofen and compounds 5a-g to have weaker affinities to COX-2 binding site. Noteworthy, the docking showed one unfavorable interaction between the hydrazide

moiety in 5 and TYR371. However, there could be one favorable hydrogen bond in common between compound 5 and celecoxib with ARG106 (Figure S9.).

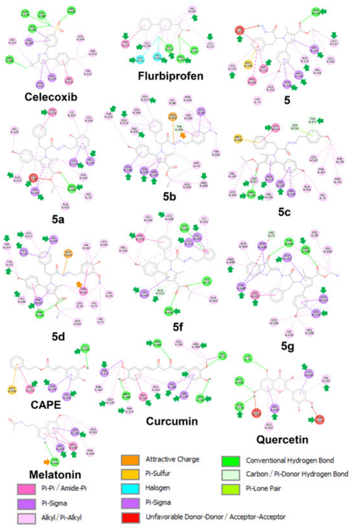

Figure S9. Interaction of the test compounds, co-crystallized ligand controls, known inhibitor controls and known natural compounds with pleiotropic antioxidant effects with COX-2. Arrows represent shared amino acid residue interactions with controls

COX-1

None of the tested compounds 5-5g were predicted to have any affinity to COX-1. The tested natural compounds had high predicted affinities with similar predicted binding energy values towards COX-1 active centre, only slightly lower than flurbiprofen's (Figure S4.).

CYP2E1

All of the tested compounds 5-5g had positive predicted binding energies and were predicted not to have any affinity to Cyp2E1 at all. Among the tested structures only controls had affinity to CYP2E1 and to some extent, CAPE, although with an unfavorable binding energy (Figure S4.).

Predictive variable – in vitro outcome selection

Table S4. Selected predictors by three methods, based on lasso (L), Huber robust regression (R), and random forest (RF). Predictor variables selected by a consensus of three methods were presented in blue and those selected by two methods – in orange.

Outcome	Predictor	L	R	RF
SH.SY5Y_protect_0.01	**tpsa**	Yes	No	Yes
	X.h.bond_donors	No	No	Yes
HepG2_protect_0.1	**silicos.it_logsw**	Yes	Yes	No
	**mlogp**	Yes	Yes	Yes
	**log_kp_.cm.s.**	Yes	Yes	No
	**pains_.alerts**	Yes	Yes	No
	**LOX_allost_docking_NA**	Yes	Yes	No
	**MPO_docking_NA**	Yes	Yes	No

	esol_solubility_.mol.l.	No	No	Yes
	silicos.it_solubility_.mg.ml.	No	No	Yes
SH.SY5Y_protect_0.1	**tpsa**	Yes	No	Yes
	**X.h.bond_donors**	No	Yes	Yes
SH.SY5Y_protect_0.5	X.heavy_atoms	No	No	Yes
	synthetic_accessibility	No	No	Yes
HepG2_cytotox_1	**X.rotatable_bonds**	No	Yes	Yes
	**X.h.bond_acceptors**	No	Yes	Yes
	**pains_.alerts**	No	Yes	Yes
	veber_.violations	No	No	Yes
HepG2_protect_1	**mlogp**	Yes	Yes	Yes
	**log_kp_.cm.s.**	Yes	No	Yes
SH.SY5Y_cytotox_1	**X.h.bond_acceptors**	Yes	Yes	No
	**X.h.bond_donors**	Yes	Yes	Yes
	**mlogp**	Yes	Yes	No
	**LOX_allost_docking_NA**	Yes	Yes	Yes
	**MPO_docking_NA**	Yes	Yes	No
	fraction_csp3	No	No	Yes
SH.SY5Y_protect_1	mw	No	No	Yes
	X.heavy_atoms	No	No	Yes
	esol_log_s	No	No	Yes
	synthetic_accessibility	No	No	Yes
	NOX_docking_NA	No	No	Yes
HepG2_cytotox_5	**X.h.bond_acceptors**	No	Yes	Yes
	pains_.alerts	No	Yes	No
	NOX_docking_NA	No	No	Yes
SH.SY5Y_cytotox_5	**X.h.bond_acceptors**	Yes	Yes	No
	**mlogp**	Yes	Yes	Yes
	**LOX_allost_docking_NA**	Yes	Yes	Yes
	X.h.bond_donors	No	Yes	No
	MPO_docking_NA	No	Yes	No
SH.SY5Y_protect_5	mr	No	Yes	No
	silicos.it_logsw	No	Yes	No
	X.rotatable_bonds	No	No	Yes
	X.h.bond_acceptors	No	No	Yes
	esol_solubility_.mg.ml.	No	No	Yes
	esol_solubility_.mol.l.	No	No	Yes

	ali_solubility_.mg.ml.	No	No	Yes
	silicos.it_solubility_.mol.l.	No	No	Yes
	silicos.it_log_p	No	No	Yes
	bioavailability_score	No	No	Yes
	synthetic_accessibility	No	No	Yes
	NOX_docking_NA	No	No	Yes
HepG2_cytotox_10	**pains_.alerts**	Yes	Yes	Yes
	**X.h.bond_acceptors**	No	Yes	Yes
HepG2_protect_10	X.heavy_atoms	No	No	Yes
	mr	No	No	Yes
	ali_log_s	No	No	Yes
	NOX_docking_NA	No	No	Yes
SH.SY5Y_cytotox_10	muegge_.violations	Yes	No	No
	bioavailability_score	Yes	No	No
	**LOX_allost_docking_NA**	Yes	Yes	No
SH.SY5Y_protect_10	mr	No	Yes	No
	X.heavy_atoms	No	No	Yes
	X.h.bond_acceptors	No	No	Yes
	tpsa	No	No	Yes
	esol_log_s	No	No	Yes
	esol_solubility_.mg.ml.	No	No	Yes
	esol_solubility_.mol.l.	No	No	Yes
	ali_solubility_.mol.l.	No	No	Yes
	wlogp	No	No	Yes
	silicos.it_log_p	No	No	Yes
	consensus_log_p	No	No	Yes
	veber_.violations	No	No	Yes
	muegge_.violations	No	No	Yes
	bioavailability_score	No	No	Yes
	pains_.alerts	No	No	Yes
	leadlikeness_.violations	No	No	Yes
	synthetic_accessibility	No	No	Yes
	LOX_act_docking_NA	No	No	Yes
	NOX_docking_NA	No	No	Yes
HepG2_protect_20	ali_solubility_.mg.ml.	No	No	Yes
	mlogp	No	No	Yes
	consensus_log_p	No	No	Yes
	log_kp_.cm.s.	No	No	Yes

	NOX_docking_NA	No	No	Yes
SH.SY5Y_cytotox_25	silicos.it_logsw	Yes	No	No
	**mlogp**	Yes	No	Yes
	log_kp_.cm.s.	Yes	No	No
	pains_.alerts	Yes	No	No
	LOX_allost_docking_NA	Yes	No	No
	xlogp3	No	No	Yes
erythrocytes_hemolysis_25	**X.h.bond_donors**	Yes	Yes	No
	**muegge_.violations**	Yes	Yes	No
	**pains_.alerts**	Yes	Yes	No
	**LOX_act_docking_NA**	Yes	Yes	No
	**MPO_docking_NA**	Yes	Yes	No
	**NOX_docking_NA**	Yes	Yes	No
	tpsa	No	No	Yes
	Cyp_2E1_docking_NA	No	No	Yes
ABTS_antioxidant_31	**X.h.bond_donors**	Yes	Yes	No
	**log_kp_.cm.s.**	Yes	Yes	No
	**pains_.alerts**	Yes	Yes	No
	**LOX_allost_docking_NA**	Yes	Yes	No
	**MPO_docking_NA**	Yes	Yes	No
	**NOX_docking_NA**	Yes	Yes	No
DPPH_antioxidant_31	**log_kp_.cm.s.**	Yes	No	Yes
	veber_.violations	No	Yes	No
	MPO_docking_NA	No	No	Yes
HepG2_cytotox_50	**fraction_csp3**	No	Yes	Yes
	LOX_allost_docking_NA	No	Yes	No
	**MPO_docking_NA**	No	Yes	Yes
SH.SY5Y_cytotox_50	**fraction_csp3**	Yes	Yes	No
	**X.h.bond_donors**	Yes	No	Yes
	pains_.alerts	Yes	No	No
	**tpsa**	No	Yes	Yes
erythrocytes_hemolysis_50	X.h.bond_donors	No	Yes	No
	**tpsa**	No	Yes	Yes
	muegge_.violations	No	Yes	No
	LOX_act_docking_NA	No	Yes	No
	MPO_docking_NA	No	Yes	No
	NOX_docking_NA	No	Yes	No
	Cyp_2E1_docking_NA	No	No	Yes

HepG2_cytotox_75	pains_.alerts	Yes	Yes	Yes
	X.h.bond_acceptors	No	Yes	No
	mlogp	No	Yes	No
	bioavailability_score	No	Yes	No
	silicos.it_log_p	No	No	Yes
SH.SY5Y_cytotox_75	**fraction_csp3**	No	Yes	Yes
	**MPO_docking_NA**	No	Yes	Yes
HepG2_cytotox_100	**X.rotatable_bonds**	Yes	No	Yes
	**mr**	Yes	Yes	No
	ilogp	Yes	Yes	Yes
	LOX_act_docking_NA	Yes	No	No
	muegge_.violations	No	Yes	No
SH.SY5Y_cytotox_100	**fraction_csp3**	No	Yes	Yes
	**MPO_docking_NA**	No	Yes	Yes
erythrocytes_hemolysis_100	**ali_log_s**	Yes	Yes	No
	tpsa	No	Yes	No
	muegge_.violations	No	Yes	No
	pains_.alerts	No	Yes	No
	Cyp_2E1_docking_NA	No	Yes	No
	MPO_docking_NA	No	Yes	No
ABTS_antioxidant_125	log_kp_.cm.s.	Yes	Yes	Yes
	**X.h.bond_donors**	No	Yes	Yes
	silicos.it_logsw	No	Yes	No
	pains_.alerts	No	Yes	No
	brenk_.alerts	No	Yes	No
	MPO_docking_NA	No	Yes	No
	NOX_docking_NA	No	Yes	No
	X.aromatic_heavy_atoms	No	No	Yes
	silicos.it_solubility_.mg.ml.	No	No	Yes
	silicos.it_solubility_.mol.l.	No	No	Yes
	mlogp	No	No	Yes
	lipinski_.violations	No	No	Yes
	bioavailability_score	No	No	Yes
	leadlikeness_.violations	No	No	Yes
DPPH_antioxidant_125	**log_kp_.cm.s.**	Yes	No	Yes
	veber_.violations	No	Yes	No
	mlogp	No	No	Yes
	MPO_docking_NA	No	No	Yes
ABTS_antioxidant_250	**X.h.bond_donors**	No	Yes	Yes

	**log_kp_.cm.s.**	No	Yes	Yes
	LOX_allost_docking_NA	No	Yes	No
	MPO_docking_NA	No	Yes	No
	NOX_docking_NA	No	Yes	No
	esol_log_s	No	No	Yes
	xlogp3	No	No	Yes
	mlogp	No	No	Yes
DPPH_antioxidant_250	**log_kp_.cm.s.**	Yes	No	Yes
	mlogp	No	No	Yes
HepG2_cytotox_250	**mlogp**	No	Yes	Yes
	esol_log_s	No	No	Yes
	esol_solubility_.mol.l.	No	No	Yes
	ali_log_s	No	No	Yes
	silicos.it_logsw	No	No	Yes
	silicos.it_solubility_.mol.l.	No	No	Yes
	xlogp3	No	No	Yes
	synthetic_accessibility	No	No	Yes

S1 References

1. Tzankova, D.; Vladimirova, S.; Aluani, D.; Yordanov, Y.; Peikova, L.; Georgieva, M. Synthesis, Safety and Antioxidant Activity of New Pyrrole Hydrazones. *Acta Pharm.* **2020**, *70*, 303–324, doi:10.2478/acph-2020-0026.
2. Tzankova, D.; Kuteva, H.; Mateev, E.; Stefanova, D.; Dzhemadan, A.; Yordanov, Y.; Mateeva, A.; Tzankova, V.; Kondeva-Burdina, M.; Zlatkov, A.; et al. Synthesis, DFT Study, and In Vitro Evaluation of Antioxidant Properties and Cytotoxic and Cytoprotective Effects of New Hydrazones on SH-SY5Y Neuroblastoma Cell Lines. *Pharmaceuticals* 2023, *16*.
3. Glur, C. *Data.Tree: General Purpose Hierarchical Data Structure*; 2023;
4. Iannone, R.; Roy, O. *DiagrammeR: Graph/Network Visualization*; 2024;
5. Cortes, C.; Vapnik, V. Support-Vector Networks. *Mach. Learn.* **1995**, *20*, 273–297, doi:10.1007/BF00994018.
6. Daina, A.; Michielin, O.; Zoete, V. SwissADME: A Free Web Tool to Evaluate Pharmacokinetics, Drug-Likeness and Medicinal Chemistry Friendliness of Small Molecules. *Sci. Rep.* **2017**, *7*, 42717, doi:10.1038/srep42717.
7. Daina, A.; Zoete, V. A BOILED-Egg To Predict Gastrointestinal Absorption and Brain Penetration of Small Molecules. *Chemmedchem* **2016**, *11*, 1117–1121, doi:10.1002/cmdc.201600182.
8. Irwin, J.J.; Sterling, T.; Mysinger, M.M.; Bolstad, E.S.; Coleman, R.G. ZINC: A Free Tool to Discover Chemistry for Biology. *J. Chem. Inf. Model.* **2012**, *52*, 1757–1768, doi:10.1021/ci3001277.
9. O'Boyle, N.M.; Banck, M.; James, C.A.; Morley, C.; Vandermeersch, T.; Hutchison, G.R. Open Babel: An Open Chemical Toolbox. *J. Cheminformatics* **2011**, *3*, 33, doi:10.1186/1758-2946-3-33.
10. Berman, H.M.; Westbrook, J.; Feng, Z.; Gilliland, G.; Bhat, T.N.; Weissig, H.; Shindyalov, I.N.; Bourne, P.E. The Protein Data Bank. *Nucleic Acids Res.* **2000**, *28*, 235–242, doi:10.1093/nar/28.1.235.

11. Kamali, A.; Ziadlou, R.; Lang, G.; Pfannkuche, J.; Cui, S.; Li, Z.; Richards, R.G.; Alini, M.; Grad, S. Small Molecule-Based Treatment Approaches for Intervertebral Disc Degeneration: Current Options and Future Directions. *Theranostics* **2021**, *11*, 27–47, doi:10.7150/thno.48987.
12. Trott, O.; Olson, A.J. AutoDock Vina: Improving the Speed and Accuracy of Docking with a New Scoring Function, Efficient Optimization and Multithreading. *J. Comput. Chem.* **2010**, *31*, 455–461, doi:10.1002/jcc.21334.
13. Eberhardt, J.; Santos-Martins, D.; Tillack, A.F.; Forli, S. AutoDock Vina 1.2.0: New Docking Methods, Expanded Force FIeld, and Python Bindings. **2021**, 7.
14. Lountos, G.T., Jiang, R., Wellborn, W.B., Thaler, T.L., Bommarius, A.S., Orville, A.M. The Crystal Structure of Water-Forming NAD(P)H Oxidase from Lactobacillus Sanfranciscensis. **2006**, doi:10.2210/pdb2cdu/pdb.
15. Forbes, L.V., Sjogren, T., Auchere, F., Jenkins, D.W., Thong, B., Laughton, D., Hemsley, P., Pairaudeau, G., Eriksson, H., Unitt, J.F., Kettle, A.J. Myeloperoxidase in Complex with the Revesible Inhibitor HX1. **2013**, doi:10.1074/JBC.M113.507756.
16. Meneely, K.M., Porubsky, P.R., Scott, E.E. Human Cytochrome P450 2E1 in Complex with the Inhibitor 4-Methylpyrazole. **2008**, doi:10.2210/pdb3e4e/pdb.
17. Newcomer, M.E., Gilbert, N.C., Neau, D.B. The Structure of Stable-5-Lipoxygenase Bound to NDGA. **2020**, doi:10.2210/pdb6n2w/pdb.
18. Newcomer, M.E., Gilbert, N.C., Neau, D.B. The Structure of Stable-5-Lipoxygenase Bound to AKBA. **2020**, doi:10.2210/pdb6ncf/pdb.
19. Kiefer, J.R., Kurumbail, R.G., Stallings, W.C., Pawlitz, J.L. Structure of Celecoxib Bound at the COX-2 Active Site. **2010**, doi:10.2210/pdb3ln1/pdb.
20. Sidhu, R.S. Crystal Structure of Cyclooxygenase-1 in Complex with Flurbiprofen. **2010**, doi:10.2210/pdb3n8z/pdb.
21. Wickham, H. *Ggplot2: Elegant Graphics for Data Analysis*; Springer Science & Business Media, 2009; ISBN 978-0-387-98141-3.
22. Posit team *RStudio: Integrated Development Environment for R*; Posit Software, PBC: Boston, MA, 2025;
23. R Core Team *R: A Language and Environment for Statistical Computing*; R Foundation for Statistical Computing: Vienna, Austria, 2025;
24. BIOVIA Discovery Studio Visualiser 2021 (version 21.1.0.20298). Available online: https://discover.3ds.com/discovery-studio-visualizer-download (accessed on 06.08.2021)
